# Digital infrastructure to support and share daily high-throughput experimental materials research

**DOI:** 10.1039/d6fd00019c

**Published:** 2026-08-28

**Authors:** Lena A. Mittmann, Hampus Nässtrom, Eugène Bertin, Sarthak Kapoor, Inês Diogo, Anat Itzhak, Giulia Dalmonte, Rasmus Thorup Danielsen, Malthe Emil Skytte, Holger von Wenckstern, Andrea Crovetto, José A. Márquez

**Affiliations:** a National Centre for Nano Fabrication and Characterization (DTU Nanolab), Technical University of Denmark 2800 Kongens Lyngby Denmark mittma@dtu.dk; b Department of Physics and CSMB, Humboldt-Universität zu Berlin 10117 Berlin Germany hampus.naesstroem@physik.hu-berlin.de; c Felix Bloch Institute for Solid State Physics, Leipzig University 04103 Leipzig Germany

## Abstract

High-throughput and autonomous experimentation generate complex, heterogeneous datasets whose reuse, large-scale analysis, and linking to data from first-principles calculations remain challenging. Here, we report on an operational deployment of the open-source NOMAD Oasis data infrastructure as a digital backbone for daily high-throughput experimental work in a materials discovery group at DTU Nanolab. The customized platform supports the complete experimental workflow in a thin-film laboratory, linking physical inventory items, automated synthesis logs, high-throughput characterization data, and cloud-based analysis within a unified data model. The platform preserves full sample provenance across multi-step workflows and uses unified sample coordinates to enable direct cross-technique correlation. We demonstrate the capabilities of the platform on a combined experimental–computational project centered on phosphosulfide thin-film libraries. The data consists of 12 000 experimental samples acquired during routine operation over 18 months, complemented by more than 800 first-principles calculations – all managed in a unified data platform available at https://nomad.nanolab.dtu.dk/nomad-oasis/gui/search/combinatorial-samples. We show how an open, extensible data infrastructure system supports reproducible workflows, scalable analysis, and long-term data reuse.

## Abstract 2

We report on an operational deployment of the open-source NOMAD Oasis data infrastructure as a digital backbone for daily high-throughput experimental work in a materials discovery group at DTU Nanolab.

## Graphical abstract

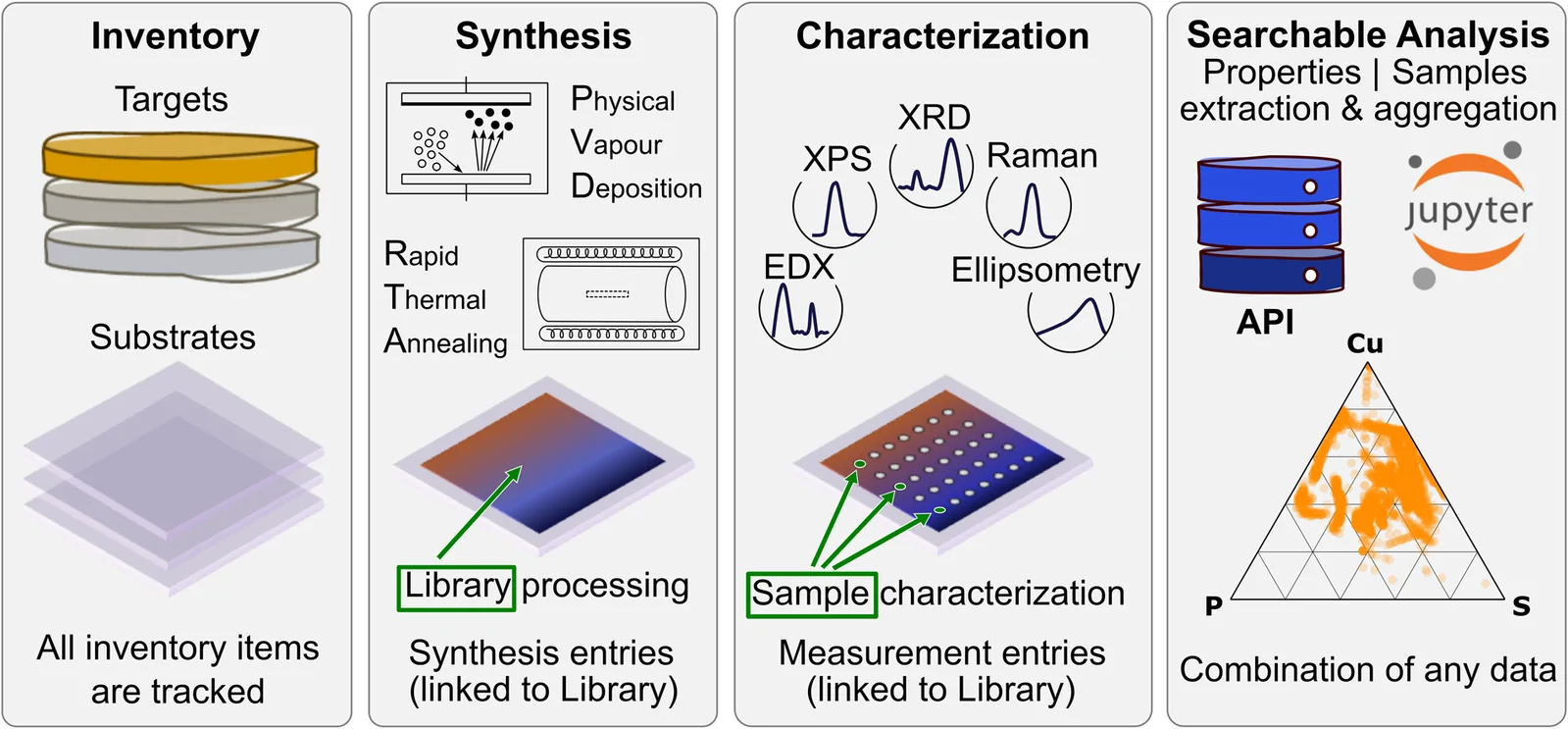

## Introduction

Automated and autonomous experimental techniques in materials research are now enabling synthesis and characterization of thousands of physical samples every day.^1–3^ Appropriate data infrastructure for experimental materials science^4–10^ has become essential to avoid data management and analysis becoming the rate-limiting steps of accelerated materials research. Two dilemmas emerge when designing such data infrastructure systems: locality *versus* globality, and customization *versus* standardization.

On the one hand, the infrastructure should be practical and convenient for the daily fieldwork of scientists in a research team (locality). It should ideally include electronic lab notebooks (ELNs), convenient handling of synthesis and characterization data files, built-in visualization, and semi-automated analysis tools that avoid repetition of tedious tasks. On the other hand, the locally produced data should be openly accessible – and understandable – to the public according to the widely accepted FAIR principles of findable, accessible, interoperable, and reusable data (globality).^11^ Global availability of machine-readable experimental data from both “successful” and “failed” experiments would enable harnessing the large portion of materials research data that would otherwise never be published. This has far-reaching benefits for training large AI models and informing researchers on, *e.g.*, previous unsuccessful attempts to synthesize new materials.^12^

The second dilemma (customization *versus* standardization) is driven by the rich diversity of methods, processes, equipment, and data types characteristic of experimental materials science. This contrasts with computational materials science, where factors such as more controlled simulation environments, a smaller set of simulation programs, and inherently digital data, make it less challenging to enforce standardization. The priority of local experimental teams is usually to tailor the data infrastructure to their own synthesis and characterization routines (customization). These are usually influenced by the desired material form (single crystals, thin films, nanoparticles *etc.*), material family (ceramics, intermetallics, semiconductors, polymers *etc.*), properties of interest, and available equipment. Conversely, database providers and data scientists benefit from putting data from different research labs and materials science domains “in the same box” (standardization). Typical reasons include database reusability, data interoperability, and ease of retrieval. Ultimately, standardization holds immense potential for the whole materials science community. It would enable comprehensive experimental databases of processes and properties, as well as more seamless integration of experimental and simulated data.

Within the current generation of openly publicized data infrastructure systems, we are not aware of a solution that convincingly addresses both dilemmas. The system proposed by Talley *et al.*^4^ and used to build the publicly accessible HTEM database^13^ has pioneered web- and API-based access to large amounts of otherwise unpublished experiments across many material systems. However, it is not based on open-source software, it is not easily transferable to other research groups, the database does not accept data from external researchers, and continuity of the public side of the project is unclear. An open-source infrastructure system promising wider transferability in the experimental materials science community has recently been published and made available.^5^

Nevertheless, it is still difficult to assess its usability and potential for standardization based on the publicly available data and documentation. Orchestration software for autonomous labs^14,15^ has so far focused much more on local customization than standardization and global research data availability. While multi-institutional, distributed data infrastructure systems have been deployed,^16–18^ the data is often not fully accessible to the public, is limited to high-level summaries that represent only a fraction of the generated experimental information, or does not allow external research groups to contribute their own data. For a detailed comparison between NOMAD Oasis and other research data infrastructures, see Table S3.

Here, we present the status of an ongoing effort to develop and operate a local, customized research data infrastructure at DTU Nanolab based on the extensible schema definitions of the open-source NOMAD platform (see a glossary of technical terms in Table S1). The infrastructure is designed such that any desired portion of locally generated experimental data can be made publicly available in a machine-readable form according to the FAIR data principles as part of a federated network. As a research case study, we focus on the high-throughput discovery of chemically diverse multi-anion thin-film materials using combinatorial synthesis and characterization methods,^3^ as well as density functional theory (DFT) calculations.^19–21^

Beyond demonstrating the functionality of our specific laboratory deployment, we release all data models, schema extensions, and documentation (https://dtu-nanolab-materials-discovery.github.io/nomad-dtu-nanolab-plugin/) developed in this work as open-source repositories, enabling straightforward reuse and customization by other experimental laboratories. Throughout this paper, we illustrate how our infrastructure is used daily to support experimental planning, automated data processing, cross-technique data integration, and scalable analysis, thereby functioning as a digital backbone for routine high-throughput experimental materials research.

## Results and discussion

### NOMAD Oasis as a digital backbone for experimental workflows

In this work, we present an operational NOMAD Oasis as a locally hosted deployment of the open-source NOMAD data infrastructure,^8^ developed in parallel with the physical experimental infrastructure of a high-throughput thin-film laboratory at DTU Nanolab. For clarity, terminology related to the NOMAD data infrastructure (*e.g.*, NOMAD entries, schemas, parsers, and plugins) is defined in Table S1 in the SI, and experiment-related terminology is explained in Table S2.

**Fig. 1:**
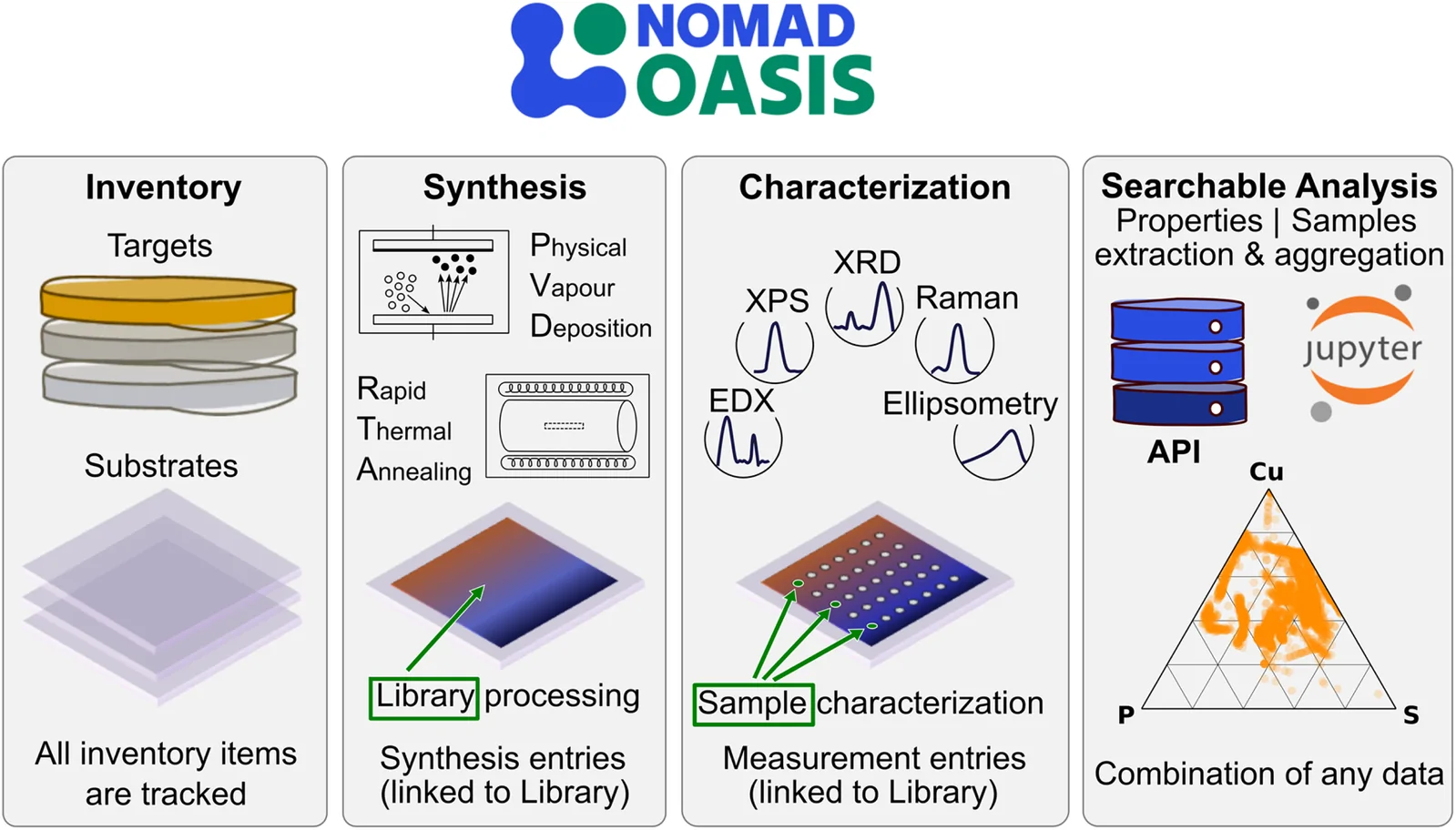
NOMAD Oasis as a digital backbone for daily high-throughput experimentation. Physical inventory items (*e.g.*, targets, substrates) are tracked and referenced by experimental processes. Synthesis workflows create and evolve combinatorial libraries with defined geometry and coordinates. High-throughput mapping measurements attach characterization data to these coordinates and thereby define sample-level entities (measurement positions) that can be aggregated across techniques. Cloud-based analysis and search then enable scalable querying and reuse of synthesis, characterization, and derived properties across projects and users.

We designed the digital infrastructure alongside the laboratory workflows, so that data capture, structuring, and reuse become an integral part of daily experimental practice. This parallel development allows the NOMAD Oasis to function as a digital backbone of the lab, tightly coupled to routine synthesis, characterization, and analysis activities (Fig. 1). Furthermore, it links experimental results to material properties calculated by first principles methods in a unified data management system.

Rather than treating data files as isolated entities, the infrastructure links objects, processes, and measurement results into coherent workflows. This organization enables full provenance tracking and cross-technique correlation while remaining compatible with the time constraints and practices of daily experimental operation.

The installation is customized through a dedicated NOMAD plugin, released as an open-source repository alongside this work. This plugin encapsulates all laboratory-specific extensions, including data schemas and automated parsers (see glossary in Table S1).^22^ Schemas provide formal, machine-readable definitions of the objects, processes, and results relevant to the laboratory, while parsers automate data handling from instrument-generated files. Upon uploading, parsers detect the file type, extract metadata and measured data, populate the corresponding schemas, and generate standardized representations and visualizations. In this way, raw synthesis logs and characterization outputs are transformed into structured, searchable records without additional manual intervention.

To balance laboratory-specific requirements with broader interoperability, we deliberately build on data models already adopted by the community^23,24^ and extend them only where necessary. For example, we represent our combinatorial sputter-deposition workflows as specializations of general physical vapor deposition (PVD) concepts, rather than introducing entirely new structures (see glossary of experimental terms in Table S2). In this way, local adaptations remain aligned with broader community models and can contribute back to future harmonization efforts, creating a balance between the strive for customization and standardization. In some cases, the inability to extract data from files with proprietary formats inhibits us from directly complying with required properties found in community standards like NeXus (see glossary in Table S1). In these cases, we implement initial data models and parsers that enable us to extract manually exported key parameters with the intention of later extending these data models to align with said community standards.

At the data-model level, the infrastructure represents physical laboratory objects and experimental activities explicitly. Inventory items such as sputter targets, substrates, gas supplies, and instruments are treated as persistent objects (entities) whose identity and history are preserved across experiments. Experimental actions, or activities (thin-film deposition, thermal processing, characterization measurements, data analysis, *etc.*) are captured as processes that reference these objects and generate new material states. This object- and process-centric representation ensures that synthesis and characterization data are contextualized by the physical conditions under which they were produced, allowing experimentalists to retrieve and interpret results without relying on informal notes, naming conventions, or external bookkeeping. The entity-activity approach of the data model is based on the Basic Formal Ontology (BFO) and its specialization in the Ontology for Biomedical Investigation (OBI) and Chemical Methods Ontology (CHMO).^25–27^ For details, see the data model overview in Fig. S1 and the detailed explanation of all schemas in our NOMAD plugin's documentation.^22^

In our current sputtering workflows, we typically produce spatially graded combinatorial libraries rather than uniform films (see Fig. 1 and Table S2).^20^ Accordingly, the combinatorial library is treated as the primary material entity created by a synthesis process. Individual material entities with well-defined composition, structure, and properties emerge later as samples, which we define as virtual, coordinate-anchored points on a library where material properties are characterized (Fig. 1). Thus, in this article a “sample” does not refer to a substrate coated with a film, as normally understood by thin-film scientists. The distinction between libraries and sample-level entities is essential for handling mapping measurements, correlating data across techniques, and preserving provenance in multi-step workflows. By anchoring all measurements to shared coordinate systems, the infrastructure enables direct comparison of synthesis parameters, composition, structure, and properties without manual alignment or post-processing. In the following sections, we illustrate how this digital backbone is leveraged in practice, using combinatorial thin-film research as a case study. A detailed view of our workflow is shown in Fig. S2.

### Inventory tracking

**Fig. 2:**
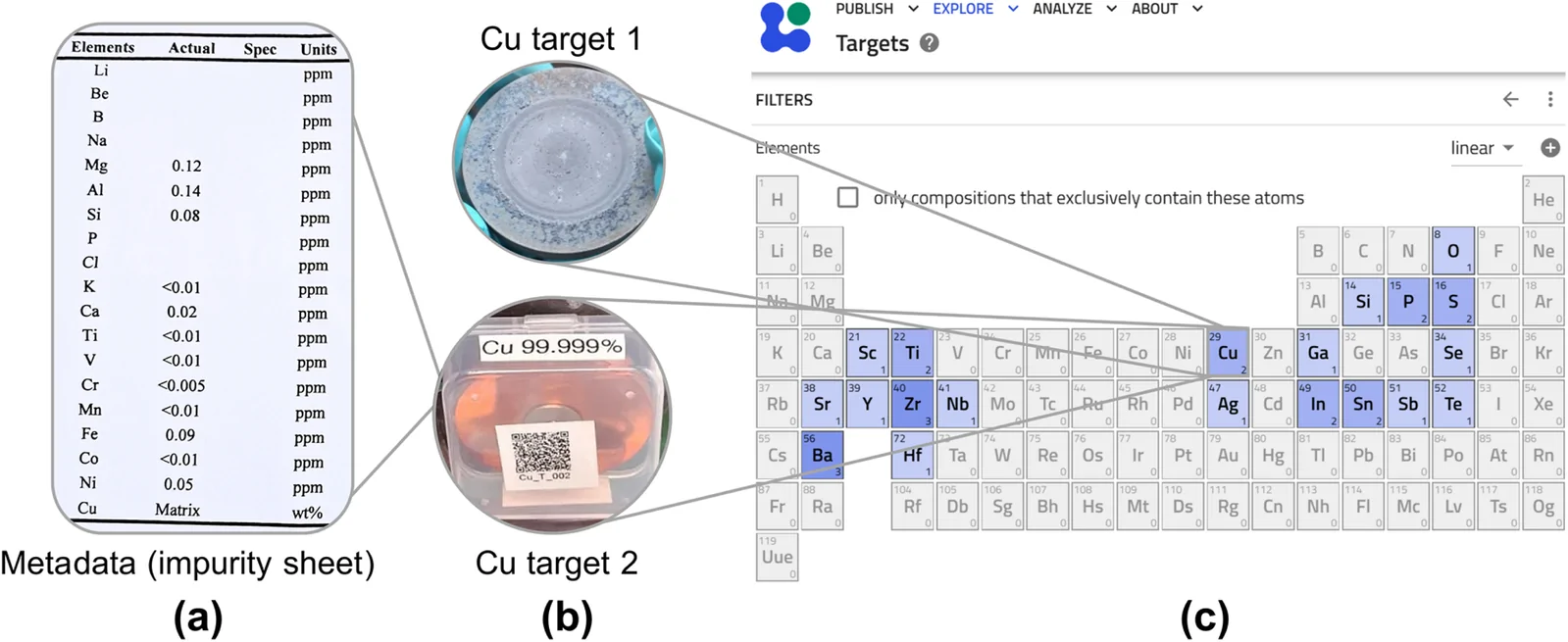
Inventory tracking in the NOMAD Oasis illustrated using sputter targets. (a) Paper-based impurity sheet from a commercial sputter target, captured and stored as standardized metadata. (b) Indexed Cu sputter targets labeled with unique identifiers and QR codes, enabling direct access to their digital records from the laboratory. (c) Web-based search interface of the NOMAD Oasis, allowing targets to be filtered by composition and metadata.

One foundational functionality of our NOMAD Oasis is to keep track of our laboratory inventory. We illustrate this functionality using sputter targets as an example (Fig. 2; https://nomad.nanolab.dtu.dk/nomad-oasis/gui/search/sputtering-targets). When purchasing a sputter target, suppliers typically provide an impurity sheet as physical or digital metadata. To make this information machine-readable and searchable, we photograph physical impurity sheets and apply optical character recognition^28,29^ to extract the relevant information into a text file, with the possibility for manual correction before upload to the NOMAD Oasis (Fig. 1).

Upon upload, the system populates a NOMAD entry and creates a corresponding QR code, which is printed and attached to the target's storage box in the laboratory. Scanning the QR code provides direct access to the target's digital record, including its composition, impurity information, and usage history.

**Fig. 3:**
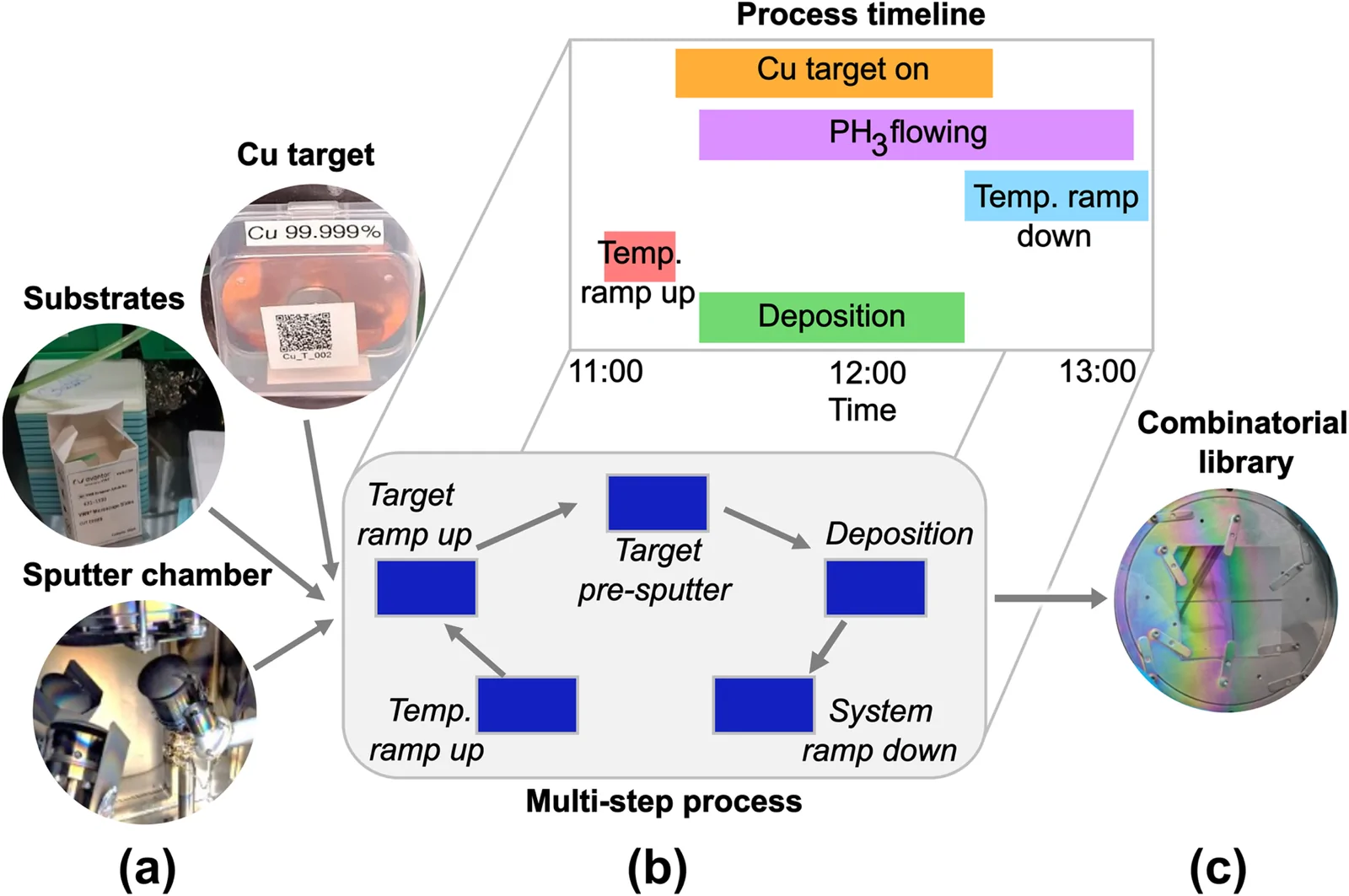
Representation of a sputter-deposition synthesis workflow in the NOMAD Oasis. (a) Physical inputs to the synthesis, including sputter targets, substrates, and deposition equipment, are represented as inventory NOMAD entries and referenced by the synthesis process. (b) A time-resolved synthesis process NOMAD entry gives structure to instrument log data, by organizing it into physically meaningful process steps (*e.g.*, temperature ramps, pre-sputtering, deposition) with associated machine signals and derived parameters. (c) The synthesis process generates one or more combinatorial library NOMAD entries, representing the deposited thin-film libraries with defined geometry and coordinate systems, which serve as the basis for subsequent characterization and analysis.

Whenever a target is mounted in the sputter deposition chamber, its identifier is entered into the proprietary control software of the deposition system. This establishes an automatic link between the deposition process log files and the target used (Fig. 3). As a result, all synthesis processes reference the corresponding target entry, enabling systematic tracking of target usage, exposure to reactive gases, and involvement in specific synthesis campaigns. A dedicated target search application allows experimentalists to quickly identify available targets and inspect their metadata and history (Fig. S6).

In the same way, substrates, combinatorial libraries, deposition instruments, evaporation materials, and reactive gases are represented as explicit digital objects within the NOMAD Oasis. These objects are linked consistently across synthesis and characterization workflows (Fig. S2), enabling coherent provenance tracking throughout routine laboratory operation. A substantial advantage for the daily work of local experimentalists is that arbitrary amounts of indistinguishable digital objects can be created in a single batch and then made distinguishable upon physical usage. This saves hours of tedious work in creating digital twins of consumables (such as substrates) that can be experimentally utilized at a rate over 10^2^/month.

### Synthesis of combinatorial libraries

Reproducibility and reuse of thin-film synthesis data remain challenging because reported process parameters are often incomplete, tool-specific, or insufficiently contextualized to be transferable across laboratories. In our NOMAD Oasis, we address this challenge by representing each synthesis run as a structured process NOMAD entry (defined in Table S1). These entries capture both time-resolved machine data and the physical context of the experiment. In addition, they explicitly link the synthesis process to the combinatorial library entities used in the process, which are created as NOMAD entries at the same time as the process entry. This approach integrates synthesis details directly into the experimental database, rather than treating them as metadata that is either lost or, at best, attached to experimental material properties.

Fig. 3 provides an overview of how physical inputs, synthesis processes, and resulting combinatorial libraries are connected within the NOMAD Oasis. A key design principle was to provide comprehensive documentation of process details with minimal effort for the experimentalists. In the current implementation, we support two thin-film synthesis methods used routinely in our lab: sputter deposition (https://nomad.nanolab.dtu.dk/nomad-oasis/gui/search/sputtering) and rapid thermal annealing (https://nomad.nanolab.dtu.dk/nomad-oasis/gui/search/rtp), or RTA (Table S2). For both methods, the corresponding NOMAD schemas (Table S1) are populated automatically from instrument-generated log files *via* dedicated parsers integrated into the NOMAD Oasis plugin.

Each sputter deposition run produces a synthesis process NOMAD entry that references all relevant physical inputs, including the sputter chamber, mounted targets, substrates, reactive gases, and auxiliary sources (Fig. 3a). During the synthesis run, the proprietary control software logs more than a hundred machine signals (such as pressures, applied powers, gas flow rates, temperatures, and target voltages) all as functions of time. After uploading the raw log files, the parser restructures them into physically meaningful process steps (*e.g.*, temperature ramps, pre-sputtering, deposition, and system ramp down), automatically identifying key events in multi-step processes (Fig. 3b).

**Fig. 4:**
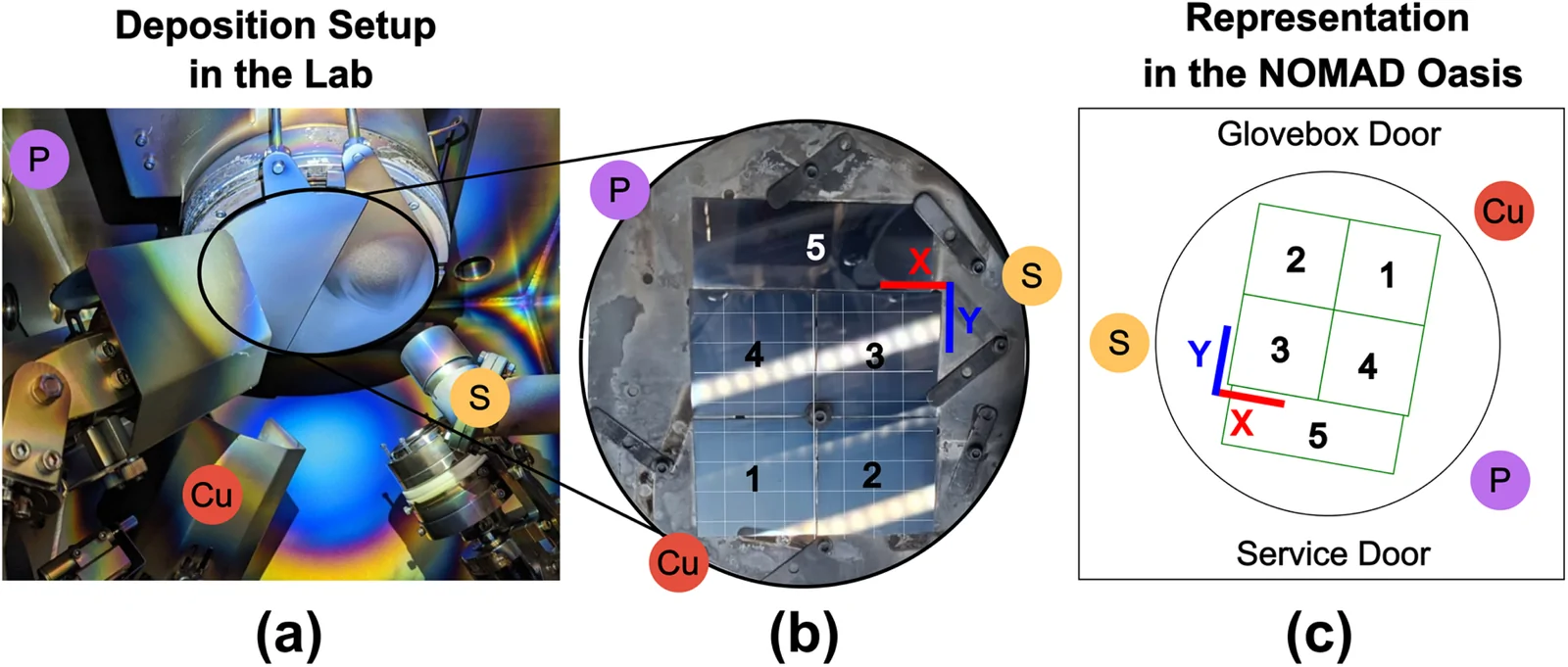
Mapping between the physical sputter-deposition setup and its representation in the NOMAD Oasis. (a) Photograph of the interior of the sputter chamber, indicating the positions of the Cu target, sulfur cracker S, and PH_3_ gas inlet. (b) Photograph of the substrate holder with five mounted substrates, annotated with their relative positions during deposition and the coordinate grid used for tracking. (c) Schematic representation of substrate and source positions as recorded and visualized in the NOMAD Oasis during a deposition run.

From these time-resolved traces, the system derives summary quantities that characterize the synthesis conditions, such as the average deposition pressure or substrate temperature during the deposition step, to provide a quick overview to the experimentalist. The parsed data and derived parameters are written into the sputtering schema and presented on the NOMAD Oasis web interface, where users can inspect both high-level process information and detailed time-dependent traces. In addition, the NOMAD Oasis automatically generates helper plots that visualize the time evolution of process parameters and the geometric configuration of material and gas sources relative to the substrates (Fig. 4, S11, and S12). These spatially resolved visualizations are particularly important for combinatorial libraries, where deposition geometry strongly influences material composition and properties.

Each sputter deposition process entry produces one combinatorial library for each mounted substrate (Fig. 3c). A library entry links the physical substrate to the synthesis process and defines a library-centric coordinate system that is shared across subsequent characterization and analysis steps. This coordinate definition ensures that synthesis parameters, composition, structure, and properties can later be correlated directly without manual alignment or post-processing.

All synthesis process and library NOMAD entries are immediately searchable through the NOMAD Oasis web interface. A dedicated sputter process search application allows users to filter previously executed synthesis processes by parameters such as target material, gas composition, pressure ranges, or deposition geometry (Fig. S7). This makes it straightforward to revisit earlier experiments and diagnose issues that may not have been apparent in real time during the experiment, such as instabilities in target voltage, gradual changes in deposition rate due to target erosion, or systematic degradation of base pressure after exposure to specific reactive gases.

The immediate availability and searchability of synthesis data has significant advantages both locally and globally, such as systematic comparison across experiments, maintaining an overview of completed work, easing the integration of historical data when planning future experiments, and facilitating the publication of synthesis data in accordance with FAIR principles.

### Evolution of combinatorial libraries through multiple processes

**Fig. 5:**
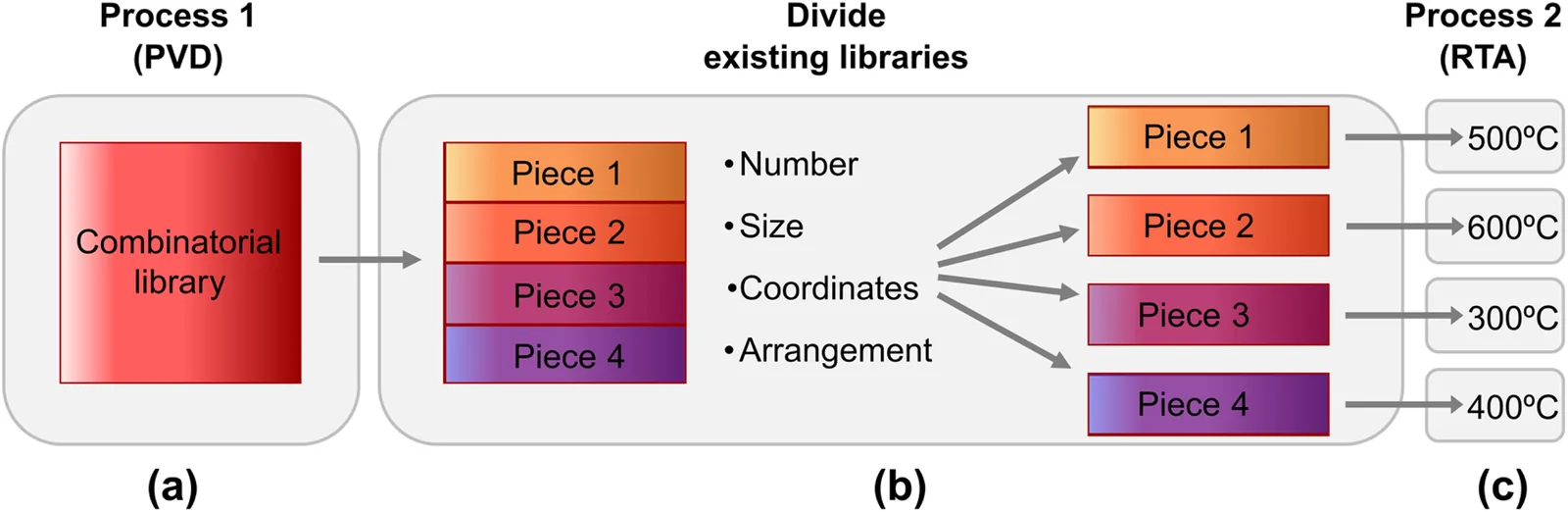
Multi-process workflow enabled by virtual library cleaving. (a) A first synthesis step (*e.g.*, PVD) generates a parent combinatorial library. (b) The library is divided into user-defined pieces; in the NOMAD Oasis, this creates child combinatorial library NOMAD entries linked to the parent while recording each piece's geometry and position/orientation relative to the parent library coordinate system. (c) Child libraries can undergo subsequent processing (*e.g.*, RTA at different temperatures) while preserving full provenance and enabling access to all previously recorded synthesis and characterization data.

Combinatorial synthesis can be greatly enhanced by mixing fragments of combinatorial libraries in additional processes that further modify the initially synthesized materials (Fig. 5 and S2). A classic example in thin-film synthesis is the initial synthesis by sputter deposition, followed by rapid thermal annealing (RTA) of selected library fragments. Digital support of such workflows requires preserving spatial provenance while allowing for flexible physical handling of samples.

To this end, we implemented virtual cleaving of combinatorial libraries in the NOMAD Oasis. In this data model, a parent combinatorial library NOMAD entry (see Fig. 5a) can be divided into an arbitrary number of child library entries with user-defined size and shape. Each child library remains linked to its parent, and its spatial position relative to the parent library is recorded (see Fig. 5b). This allows individual fragments to be processed, characterized, and analyzed independently, while retaining access to the full synthesis and characterization history of the original library.

Structurally, the RTA schema mirrors the sputtering schema introduced earlier: just as the sputtering workflow extends the physical vapor deposition (PVD) base schema, the RTA data model is implemented as an extension of the chemical vapor deposition (CVD) base schema (see definitions in Tables S1 and S2).

Moreover, the RTA instrument control software also records time-resolved signals – including temperature, heating power, chamber pressure, and gas flow rates. Because this logging strategy closely matches that of the sputtering instrument, we were able to adapt the existing parsing, feature-extraction, and visualization pipeline to handle RTA data and seamlessly display it in the web interface.

While our NOMAD Oasis currently integrates only two synthesis techniques, the architecture was intentionally designed to scale to an arbitrary number of available techniques. Each technique may comprise complex multistep processes, custom geometries, and arbitrary library placements. Different techniques can be chained together in any order to build sophisticated synthesis sequences. The reuse of definitions simplifies the implementation of new methods and ensures a seamless integration into the existing system. Together, these design choices make the deployment straightforward to extend as new synthesis methods are added, without disrupting existing workflows.

### High-throughput characterization of combinatorial libraries

We integrated a broad set of high-throughput, mapping-type characterization techniques into the NOMAD Oasis (https://nomad.nanolab.dtu.dk/nomad-oasis/gui/search/edx-measurements), including energy-dispersive X-ray spectroscopy (EDX), X-ray photoelectron spectroscopy (XPS), X-ray diffraction (XRD), spectroscopic ellipsometry, steady-state photoluminescence (PL), Raman spectroscopy, and optical reflection/transmission spectroscopy. For each supported characterization technique, we can upload raw result files (*e.g.*, optical spectra, diffraction patterns) for later analysis and property extraction, and/or instrument-exported result files that already contain processed data (*e.g.*, elemental composition, optical functions).

**Fig. 6:**
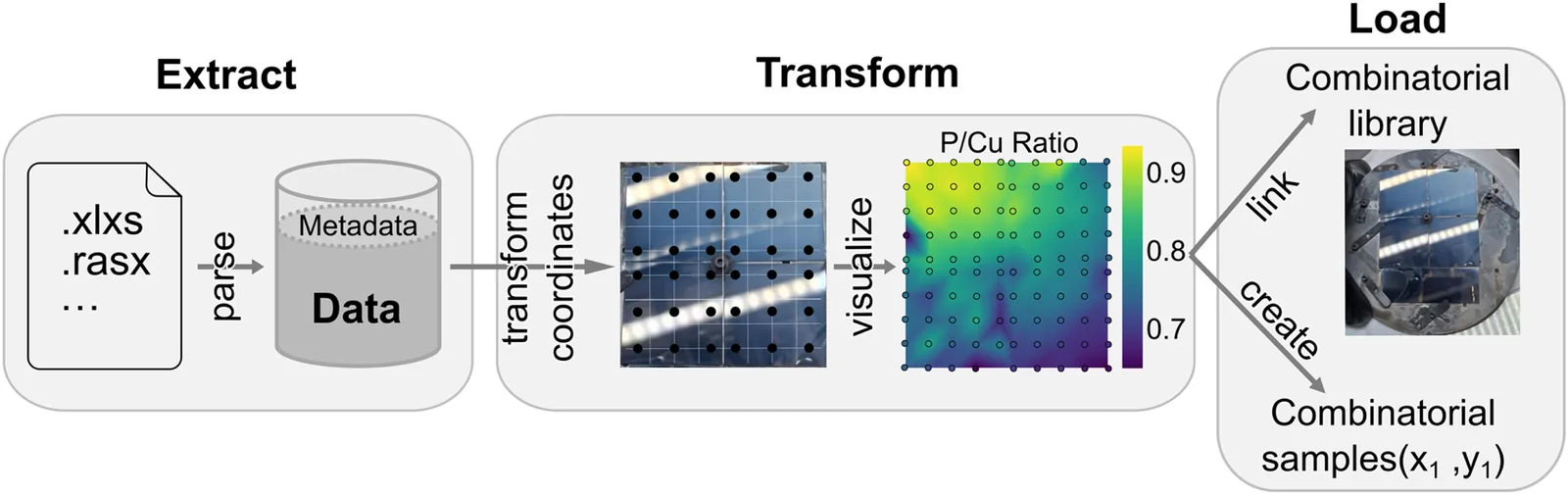
High-throughput characterization data processing in the NOMAD Oasis. Measurement files in different formats are uploaded and parsed into their respective data schemas, containing both the relevant data and the metadata describing data acquisition. A coordinate transformation is applied to the measurement point positions using calibration-point coordinates and visualizations are automatically created. The measurement data entry is linked to the combinatorial library on which it was measured, and samples at the same location are created.^30^

Dedicated parsers in NOMAD Oasis automatically convert these files into structured, searchable records while linking each measurement to the correct combinatorial library (Fig. 6). Proprietary file formats are handled just like openly accessible file formats, and their content is stored in a .json format to ensure future access.

Upon upload, the system extracts measurement data together with acquisition metadata (*e.g.*, integration time, slit settings, instrument parameters). The same pipeline handles both single-point and mapping measurements: it reads any spatial coordinates in the file and attaches them to the measured values. When measurements are performed on a grid defined in instrument-stage coordinates, the positions are automatically transformed into the library-centric coordinate system defined at library creation. This transformation uses calibration points (*e.g.*, substrate corners) that are either recorded directly in instrument coordinates or provided by the user at upload, enabling cross-technique alignment without manual post-processing.

To support rapid inspection and quality control, basic data visualization is automatically presented to the user directly after data handling. Examples include composition heat maps for EDX (Fig. S13) and waterfall plots for XRD patterns (Fig. S14), allowing users to assess coverage, outliers, and measurement consistency immediately, and enabling later reuse by collaborators or external users.

Finally, the data handling step connects characterization outputs to sample-level entities (Table S1). Each measurement position is represented as a coordinate-anchored sample on the parent library. If a sample at the same location already exists from a previous technique, the new measurement is appended to it; if not, a new sample is created. This progressively builds multi-modal sample records that aggregate multiple properties (composition, structure, optical, vibrational) at shared coordinates. In case different techniques use different measurement grids, we apply predefined utilities for positional tolerances and interpolation to associate data consistently across nearly matching positions. In this way, high-throughput characterization uploads immediately populate a coherent dataset across techniques that can be queried by synthesis parameters, measurement conditions, and derived properties (Fig. S9).

At this stage, the data models used for representing the characterization data are mostly use-case driven implementations focused on enabling the identification of key material properties. These data models can later be extended to align with community standards such as existing NeXus application definitions (see glossary in Table S1) for XPS, electron microscopy, spectroscopic ellipsometry, and Raman spectroscopy.^30^ For several of these techniques, open-source parsers are already available that translate selected instrument data into the corresponding NeXus-compliant representations, promising practical adoption of these standards in NOMAD.^31–35^

### From data to insights: integrated analysis workflows

While our NOMAD Oasis deployment provides structured capture and integration of synthesis and characterization data, extracting scientific insight from high-throughput experiments typically requires problem-specific analysis that goes beyond standard visualization. In our workflow, this analysis is performed directly within the NOMAD infrastructure using a Jupyter Hub deployed together with the NOMAD Oasis, with direct access to the mounted experimental database and installed data models. This allows analysis notebooks to operate directly on the same structured representation of synthesis processes, characterization measurements, and sample-level entities as used throughout the NOMAD Oasis (https://nomad.nanolab.dtu.dk/nomad-oasis/gui/search/analysis), without requiring manual data export or ad hoc data handling. Examples of useful tools range from loading and combining data from different libraries within the Oasis, to deriving material properties that are not accessible out of the box such as crystallite size, lattice strain, or band gap *via* batch fitting of peaks or machine learning algorithms.

Within this environment, analysis notebooks can be created through a form-based interface that allows users to select the combinatorial libraries to be analyzed. Based on these selections, the notebooks automatically construct the required application programming interface (API) queries to retrieve synthesis metadata, characterization results, and sample-level entities from the NOMAD Oasis. Because data access is handled programmatically rather than through static input files, the same notebook structure can be applied flexibly across different synthesis campaigns, projects, and time periods while always operating on the current state of the database. Additionally, the characterization results can be written back to the Oasis either as figures or as sample entries, making them permanently indexed and searchable. This enables analysis workflows to scale naturally with the growing experimental dataset and promotes synergy with community-developed open-source Python packages.

**Fig. 7:**
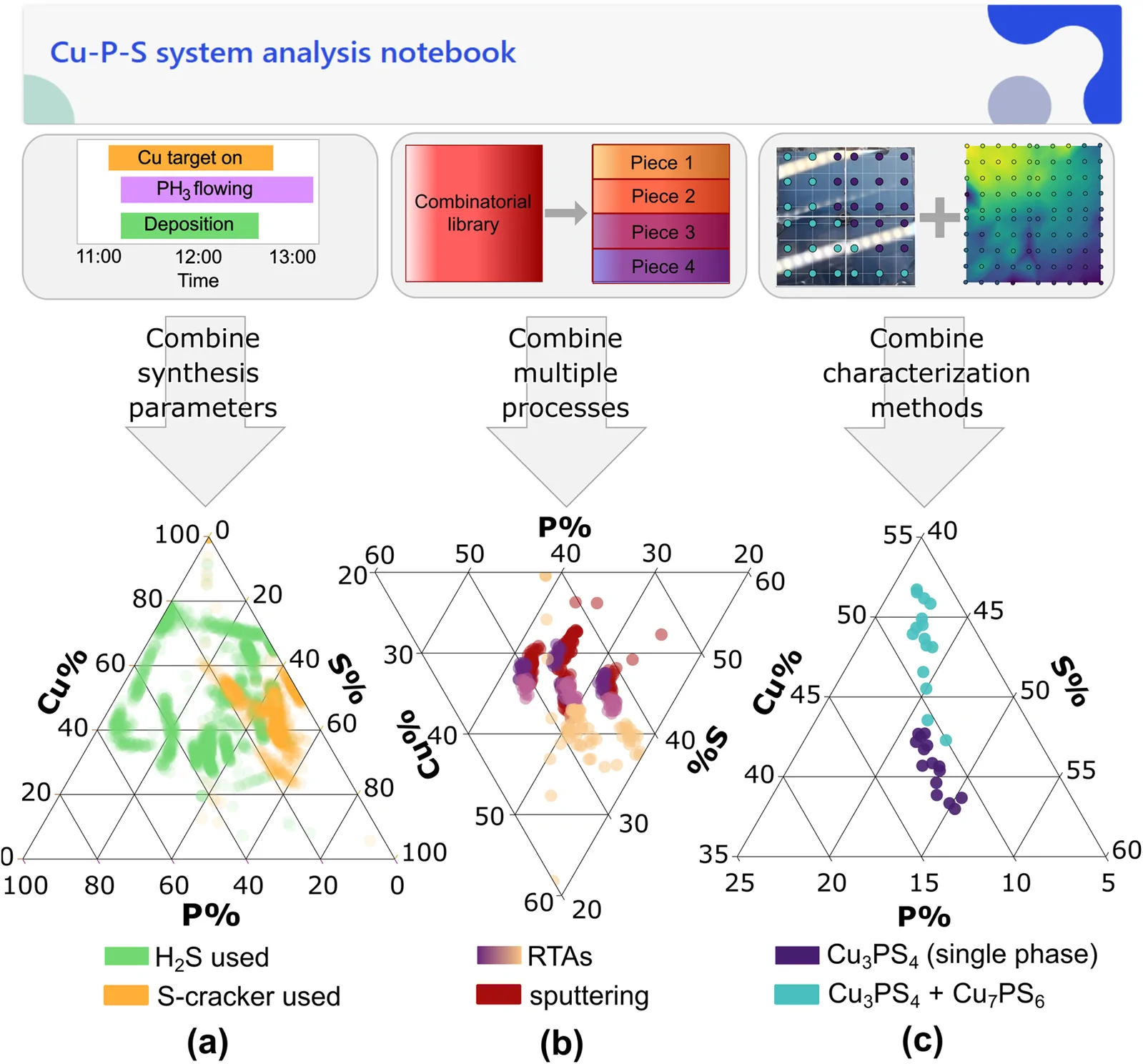
Data analysis and insights enabled by the NOMAD Oasis infrastructure. The analyses are run in templated Jupyter notebooks (header at the top) on the same server where the data is stored. (a) Combining synthesis data and characterization results to uncover trends originating from synthesis conditions. Here, the specific insight is that an S-cracker source tends to produce more sulfur-rich materials than an H_2_S source. (b) Comparing characterization results of libraries before and after additional processes. Here, the specific insight is that Cu–P–S libraries tend to lose P (and, to a lesser extent, S) after an RTA process. (c) Combining different characterization results acquired on different measurement grids by applying grid interpolation. Here, the specific insight is that a Cu_7_PS_6_ secondary phase starts precipitating from a Cu_3_PS_4_ matrix (according to XRD) when the Cu content in Cu–P–S libraries drops below 43% (according to EDX). Running the data analysis on the same servers enables direct writing of derived results from the notebooks back to the NOMAD Oasis database.

To support routine use, we create templates of frequently applied analysis workflows and make them searchable within the analysis environment. These templates implement advanced but routinely used evaluation procedures and can be easily adapted or extended. Representative instances of such templated workflows, routinely used in our daily work, are illustrated in Fig. 7.

Combining synthesis metadata with EDX-derived chemical composition can be used to visualize the compositional space accessed in films processed with different sulfur sources (Fig. 7a), allowing users to make data-driven decisions when planning future experiments. In a similar fashion, chemical composition can be directly correlated with, *e.g.*, the distance between libraries and the sputter target, enabled by consistent coordinate handling for synthesis and characterization steps. More generally, this flexibility enables the identification and analysis of trends arising from synthesis parameters that were not originally considered influential, even at later stages of a project.

As a second example, we can monitor changes in the chemical composition before and after an RTA step in a large number of samples that were initially synthesized by sputter deposition (Fig. 7b). This functionality is enabled by accurate tracking of sample provenance and coordinates through a complex synthesis and characterization history. We can perform forward analysis of library evolution through processing, as well as backward analysis to identify the reasons for success or failure to produce the desired outcome. Many other applications can be envisioned, such as aging studies of a thin film under different conditions, or the rationalization of the origin of impurities found in a later characterization, which may be due to contamination in one of the two synthesis setups caused by processing different materials in the same setup around the same time.

As a third example, Fig. 7c combines mapping data from two different characterization techniques (EDX and XRD) to investigate the boundaries of single- and multi-phase materials in composition space. Our NOMAD Oasis implementation handles all the time-consuming steps of this analysis, which would be impractical to perform manually at scale. These steps include unification of the coordinate systems of the two measurements, interpolation in cases where the measurement grids do not coincide, automated data treatment, and sample-by-sample comparison between experimental and calculated XRD patterns to assign phases or phase mixtures (Fig. S4 and S5).

Applying these types of analyses to a single library provides detailed insight into property variations in real space. Applying the same analysis across multiple libraries from various synthesis runs reveals more general material trends, such as regions of emergence of secondary phases, transitions from metallic to semiconducting behaviour, limits of incorporation of volatile elements, and dependence of all these properties on thin-film process conditions.

These exemplary analysis tasks illustrate how the data infrastructure incorporated into various aspects of everyday lab routines (Fig. 1) enables scalable and reusable analysis (Fig. 7) with exciting perspectives for future materials research.

To facilitate the reuse of these automated analysis routines, notebook-based analysis templates can be published. This enables other users to apply an existing analysis template to their own data, adapt specific components as needed and build on top of it. In this way, the complete analysis workflow from raw data to derived material properties becomes transparent, reusable, searchable, and extensible.

### Data searchability and programmatic access

When notebook-based analysis results are written back to the NOMAD Oasis as sample-level entities, analysis outcomes become persistent, indexed, and searchable data objects (https://nomad.nanolab.dtu.dk/nomad-oasis/gui/search/combinatorial-samples). Characterizing these samples using material properties (*e.g.*, composition, crystal structure, band gap) rather than raw data (*e.g.*, signal intensities, spectra) finalizes the analysis workflow and makes its outcomes persistent, indexed, and searchable.

**Fig. 8:**
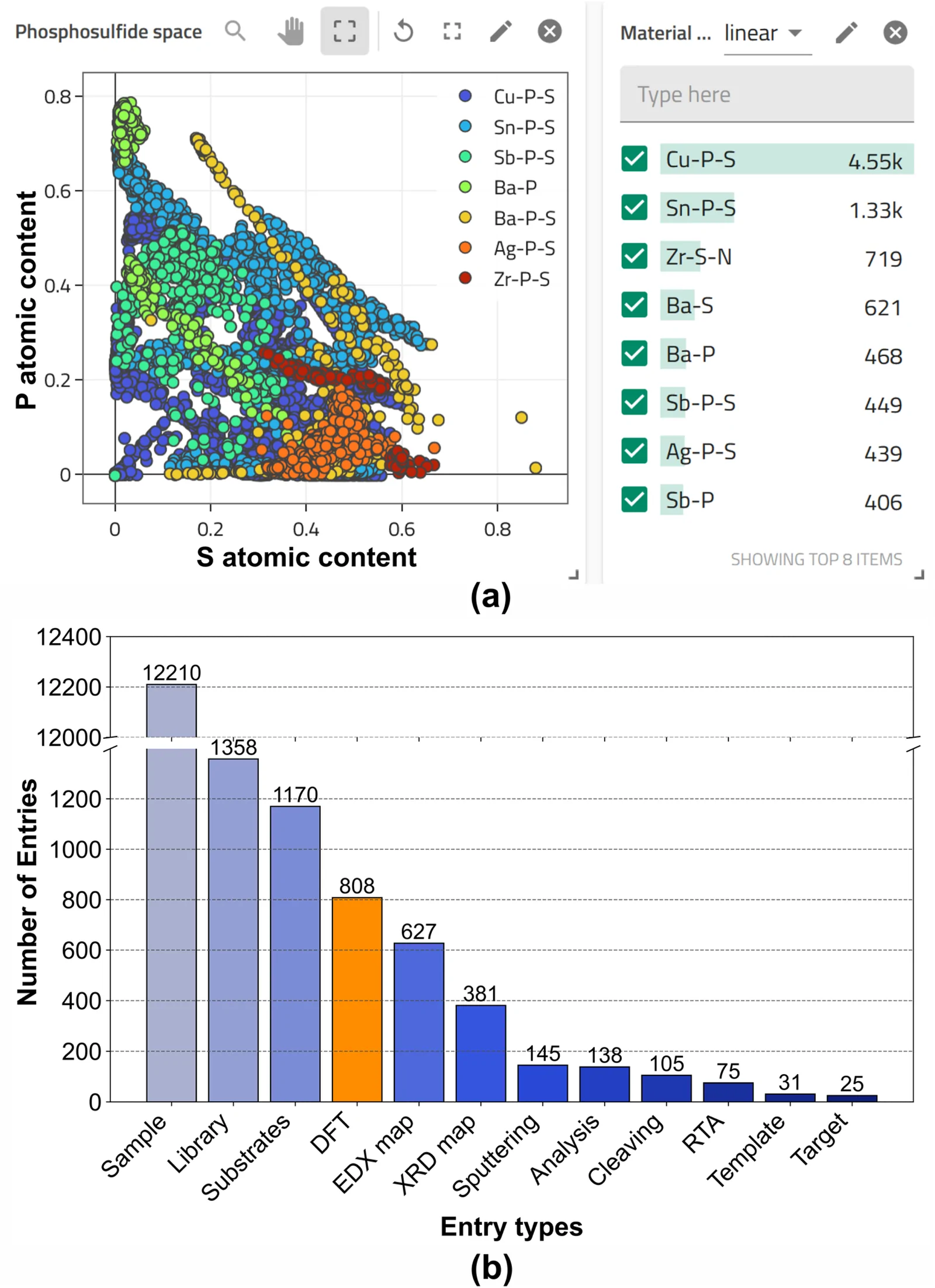
Data accessibility and searchability. (a) Screenshots of the search interface for sample entities in the NOMAD Oasis. The ∼12 000 samples can be filtered by material system (right) and visualized in an interactive plot where their position is determined by the experimentally measured sulfur and phosphorus content, and the color encodes the respective third element. This user interface enables intuitive data discovery and filtering existing data across material spaces. (b) Number of entries by entry type uploaded to the NOMAD Oasis over 18 months. Experimental entries are colored blue. First-principles calculation entries are colored orange.

We developed multiple search interfaces that allow users to query our experimental materials database based on deposition parameters, characterization outcomes, or analysis results across uploads, projects, and users (Fig. S6–S10). In particular, the search app for sample entities (Fig. 8a and S7) allows local and external researchers to search and filter by material properties in a way similar to established computational databases.^36–38^ The key advantage of an experimental database is that the influence of synthesis conditions on the resulting properties is explicit. Hence, we argue that our data infrastructure effort is a necessary step to gain access to large-scale composition–structure–process–property relationships – a holy grail of materials science.

To give an idea of the scale of our local data collection effort as of January 2026, two PhD students and two postdoctoral researchers within our group have captured full provenance and harmonized coordinates of more than 12 000 samples including 4000 individual XRD patterns in 380 maps (Fig. 8b) after routine operation for 18 months. Importantly, the NOMAD Oasis datasets include both “successful” and “failed” experiments, reflecting routine laboratory operation rather than a curated subset prepared for publication. In addition, our NOMAD Oasis integrates first-principles calculations performed within our group to complement experimental exploration; the computational dataset is accessible through its own search interface (Fig. S10) and currently contains more than 800 materials.^39^

While the graphical search interfaces enable rapid, intuitive exploration, the NOMAD Oasis also exposes all experimental and derived data through a unified application programming interface (API), enabling fully programmatic access for both local and external users. This programmatic interface supports large-scale reuse scenarios, including statistical analyses and machine-learning workflows operating directly on harmonized experimental data without manual export or format conversion, and it underpins the notebook-based analysis workflows described above.

### Sharing the data

One advantage of using NOMAD Oasis is its built-in access management system and data sharing functionality. Access is determined at the upload (*i.e.* project) level. Other users or user groups can be added as “co-authors” (read and write access) or “reviewers” (read-only access). The data can also be published, and it is then findable by anyone with access to the Oasis, either through the graphical search interface or through the API. NOMAD Oasis access is regulated on three levels: network, authentication, and authorization. Firstly, the Oasis can be restricted to a closed network (*e.g.* university intranet). Secondly, the Oasis can require users to authenticate themselves (*i.e.* log in). Finally, the authorized actions of logged in users can be controlled (*e.g.* restricting external users from anything but viewing published uploads). In summary, this means that data can safely be kept private, exchanged with specific collaborators, or shared with the public.

### Limitations and sustainability considerations

The infrastructure described here has proven effective for our research group after over two years of iterative development. We had to address numerous practical and conceptual challenges before reaching a functional deployment. The main challenges are divided into six categories: infrastructure, development, updates, documentation, data structure, and transferability. For more details, see SI Section S7.

In particular, the continuity of development in an academic work environment that is based on short-term contracts is a critical issue, as well as the interdisciplinary expertise necessary to bridge materials science, software development, and data science. This can hinder long-term use and scalability of the infrastructure. Challenges related to system maintenance and adaptability to new experimental contexts or research goals, further complicate sustainable use. While some of these issues can be mitigated through comprehensive documentation and shared responsibility among multiple contributors, they remain important constraints on robustness and reproducibility. Properly establishing, developing, maintaining, and documenting a local NOMAD Oasis such as the one described here requires considerable effort. Such development is largely driven by students and postdocs and often remains under-recognized in traditional materials science settings.

Finally, our specific NOMAD Oasis installation is not an out-of-the-box solution for other research groups. Nonetheless, the availability of its underlying software, documentation, and the high-level description given in this paper provide a solid foundation for implementation in similar research settings.

## Conclusions

We showcased a materials research data infrastructure based on the NOMAD Oasis concept. It functions as a digital backbone for daily operations in high-throughput experimental materials research in tight integration with first-principles calculations. The platform is designed to ensure easy readability by humans and machines alike, and to strike a reasonable balance between the requirements of locality, globality, customization, and standardization.

By integrating physical inventory, automated capture of synthesis processes, high-throughput characterization, and cloud-based analysis within a unified data model, we can preserve full sample provenance throughout heterogeneous synthesis processes and characterization measurements. We demonstrated direct cross-technique correlation of material properties throughout the experimental workflow, and API-accessible data structures ready to be used for training AI models. We believe our infrastructure may lay the foundations for tackling ambitious problems, such as synthesizability prediction,^40^ experiment-driven composition–structure–process–property-relationships, and inverse materials design.^41^

The infrastructure is FAIR-by-design and minimizes manual effort for experimentalists, making comprehensive data capture sustainable during routine laboratory operation. Its scalability is demonstrated by the aggregation and analysis of more than 12 000 samples acquired across multiple projects and users, including “successful” and “failed” experiments. Because the system is open-source, extensible, and based on shared data models, our approach is transferable to other laboratories, synthesis methods, and materials systems, supporting reproducible and data-driven experimental research.^42^

## Author contributions

José A. Márquez, Andrea Crovetto, Lena A. Mittmann, and Hampus Näsström conceptualized the project. Software development of the NOMAD plugin was primarily performed by Lena A. Mittmann, Hampus Näsström, and Eugène Bertin, with contributions from Sarthak Kapoor, Inês Diogo, and José A. Márquez. Data modeling was done by Lena A. Mittmann, Hampus Näsström, Eugène Bertin, Sarthak Kapoor, José A. Márquez, and Holger von Wenckstern. Software development of an initial data infrastructure prototype was conducted by Giulia Dalmonte, Rasmus T. Danielsen, and Malthe E. Skytte. Lena A. Mittmann, Anat Itzhak, Eugène Bertin, Inês Diogo, and Giulia Dalmonte collected the data present on the Oasis. José A. Márquez and Andrea Crovetto supervised the project. Lena A. Mittmann prepared the initial draft of the manuscript, with major contributions from Eugène Bertin, Andrea Crovetto, and José A. Márquez. All authors read and approved the final manuscript.

## Conflicts of interest

There are no conflicts to declare.

## Supplementary Material

FD-268-D6FD00019C-s001

## Data Availability

The NOMAD plugin repository^22^ of the NOMAD Oasis deployment at DTU Nanolab is available at: https://github.com/DTU-Nanolab-materials-discovery/nomad-dtu-nanolab-pluginhttps://doi.org/10.5281/zenodo.18417535. Comprehensive documentation of the NOMAD Oasis deployment at DTU Nanolab, covering the scope, data models, and schema definitions, is provided at: https://dtu-nanolab-materials-discovery.github.io/nomad-dtu-nanolab-plugin/. The repository containing the Docker containers and configuration files for the NOMAD Oasis deployment at DTU Nanolab is available at: https://github.com/DTU-Nanolab-materials-discovery/nomad-oasis-image. A publicly accessible NOMAD Oasis instance with a reduced dataset is available at: https://nomad.nanolab.dtu.dk/nomad-oasis/gui/search/combinatorial-samples. Supplementary information (SI) is available. See DOI: https://doi.org/10.1039/d6fd00019c.
