# Supplementary material for: Digital infrastructure to support and share daily high-throughput experimental materials research
Source: Faraday Discuss. 2026 Aug 28;268:479–99. doi: 10.1039/d6fd00019c (PMC13522971; doi:10.1039/d6fd00019c)
Supplement: Supplementary file 1 [file FD-268-D6FD00019C-s001.pdf]

Supplementary Information (SI) for Faraday Discussions.
This journal is © The Royal Society of Chemistry 2026

Supplementary information for Digital infrastructure to support and share daily high-throughput experimental materials research

Lena A. Mittmann[a,*], Hampus Nässtrom[b,*], Eugène Bertin[a], Sarthak Kapoor[b], Inês Diogo[a], Anat Itzhak[a], Giulia Dalmonte[a], Rasmus Thorup Danielsen[a], Malthe Emil Skytte[a], Holger von Wenckstern[c], Andrea Crovetto[a] and José A. Márquez[b]

a: National Centre for Nano Fabrication and Characterization (DTU Nanolab), Technical University of Denmark, 2800 Kongens Lyngby, Denmark

b: Department of Physics and CSMB Humboldt-Universität zu Berlin, 10117 Berlin, Germany

c: Felix Bloch Institute for Solid State Physics Leipzig University, 04103 Leipzig, Germany

Email: mittma@dtu.dk, hampus.naesstroem@physik.hu-berlin.de

S1. Glossaries

Table S1. Glossary of NOMAD and data infrastructure terminology

Term	Definition
**NOMAD**	An open-source, distributed research data platform for materials science that provides shared data models, storage, search, and programmatic access for experimental and computational data.
**NOMAD Oasis**	A locally hosted deployment of the NOMAD platform that allows laboratories to operate their own data infrastructure while remaining compatible with the central NOMAD ecosystem.
**Deployment / Installation**	A specific instance of NOMAD Oasis operated by a lab, including server/services, storage, configuration, and installed plugins/schemas/parsers.
**Plugin**	A modular, open-source extension of NOMAD that encapsulates laboratory-specific customizations, including data schemas and automated data handling logic.
**Schema**	A formal, machine-readable definition of the structure, metadata, and relationships of a specific type of data entry in NOMAD.
**Base schema**	A general-purpose schema provided by NOMAD that captures common concepts (e.g., physical vapor deposition) and serves as a foundation for extensions.

**Schema specialization**	A laboratory-specific extension of a base schema that adds or refines properties while remaining compatible with the original schema structure.
**API (Application Programming Interface)**	Programmatic interface to query, retrieve, and upload NOMAD entries and metadata.
**Search application (Search app)**	A web UI built on NOMAD search/indexing that provides domain-specific filters and visualizations for a set of entry types.
**Parser**	An automated component that detects uploaded data files, extracts metadata and measured values, populates the corresponding schemas, and generates standardized representations and visualizations.
**Entry**	A structured record in NOMAD corresponding to a specific object, process, measurement, or analysis result, stored according to a defined schema.
**Provenance**	The complete, traceable history of how a data entry was generated, including referenced objects, processes, parameters, and prior data.
**Jupyter Hub**	A multi-user service that authenticates users and manages isolated Jupyter notebook servers (e.g., JupyterLab) on shared infrastructure; in a NOMAD Oasis deployment, it provides users with direct, server-side access to the local database, data models, and APIs for programmatic analysis.
**Entity**	A persistent item represented in NOMAD that can be physical (target, substrate, library) or virtual/abstract (coordinate-anchored sample position), and can be referenced across processes.
**Activity**	An action to create, manipulate, or analyze entities (e.g., synthesis, annealing, characterization).

**NeXus**	NeXus is a common data format for materials science. It is being developed as an international standard by scientists and software developers representing major scientific facilities in order to facilitate consistent data exchange, analysis, and visualization across instruments and institutions.
**NeXus application definition**	NeXus application definitions define the minimum set of terms that must be used to meaningfully describe a specific type of experiment or measurement. Application definitions also specify recommended or optional terms that can be included in a NeXus data file.

Table S2. Glossary of thin-film and combinatorial workflow terminology

Term	Definition
**Combinatorial library**	A physical substrate carrying a spatially varying composition or processing gradient, treated as a single material entity created by a synthesis process.
**Sample**	A virtual, coordinate-anchored point on a combinatorial library representing a localized region where material properties are measured and aggregated across techniques.
**Mapping measurement**	A characterization measurement performed at multiple spatial positions on a library, producing property maps rather than single-point values.
**Library-centric coordinate system**	A shared spatial reference frame defined for a combinatorial library that enables alignment and correlation of measurements from different instruments.
**Sputter deposition**	A physical vapor deposition technique in which material is ejected from a target by ion bombardment in a discharge, and then deposited as a thin film on a substrate.
**Sputter target**	A solid source material used in sputter deposition from which atoms are ejected to form a thin film.
**Reactive sputtering**	A sputter deposition process performed in the presence of reactive gases to form compound thin films.
**Rapid thermal annealing (RTA)**	A thermal processing technique involving rapid heating and cooling to modify the structure and composition of thin films.

**Physical vapor deposition (PVD)**	A class of thin-film deposition techniques based on vapor-phase transport of material from a source to a substrate.
**Chemical vapor deposition (CVD)**	A vapor-phase thin-film deposition method based on chemical reactions of gaseous precursors at the substrate surface.
**Library fragment**	A physical portion of a combinatorial library obtained by cleaving or cutting, treated as a child library with preserved provenance.
**Virtual cleaving**	A data-model operation that represents the division of a parent combinatorial library into child libraries while retaining spatial relationships and processing history.

S2. Research Data Infrastructure Comparisons

Table S3. Comparison of functionality for various research data infrastructures (RDIs).

RDI	**HTEM**[4]	**Kadi4Mat**[6]	**eLabFTW**[10]	**MatInf**[5]	**datalab**[9]	**ChemOS 2.0**[14]	**AlabOS**[15]	**NOMAD Oasis**
Scope	A tightly coupled, purpose-built RDI for combinatorial inorganic thin-film materials at NREL.	A general purpose, open-source RDI for material science, focused on record linkages.	A general purpose, open-source ELN focused on experiment documentation and lab inventory/resource management	An open-source, materials science RDI focused on heterogeneous data management for high-throughput and computational data.	An open-source RDI for materials chemistry labs focused on sample and experimental/characterization management.	An open-source orchestration platform for autonomous experimental campaigns focused on instrument coordination and AI-guided design-of-experiment.	An open-source, specialized SDL framework for autonomous materials synthesis/ characterization labs	An open-source RDI for materials science. Dual focus on computational (DFT/MD) and experimental data via schema-driven MetaInfo ingestion and ELN framework.
Local deployment capability	Site-specific stack with no documented external self-deployment tooling.	Self-hostable with script-based and manual installation options. Multiple institutional instances operational.	Self-hostable with Docker and docker-compose. Several institutional instances operational.	Self-hostable codebase requiring manual server setup. Multi-tenant architecture running in multiple institutional deployments.	Self-hostable with Docker and docker-compose. Comes with Ansible and Terraform configurations for additional provisioning. Multiple institutional instances operational.	Self-hostable on local/fog hardware. Uses NixOS for reproducible, declarative environment management. Each deployment requires hardware-specific customization.	Self-hostable codebase that requires manual setup of database and message broker.	Self-hostable with Docker and docker-compose. Purpose-built for institutional self-hosting with extensive deployment documentation. Several institutional deployments operational.
Scalability	Unclear.	Runs as a system service on dedicated servers. No containerization or container orchestration.	Docker-native; supports Swarm/Kubernetes in principle. Official documentation targets single node; large scale needs manual tuning.	Multi-tenant architecture suitable for SaaS deployment across workgroups. No containerization or container orchestration.	Microservices architecture with horizontal scaling capability. Orchestration expertise required for large institutional deployments.	Not designed for multi-user, but multi-instrument scaling. Scales across distributed instruments and geographically separated lab. No containerization or container orchestration.	Single-node Python service. No containerization or multi-site horizontal scaling.	Microservices architecture with horizontal scaling capability. Terabyte-scale proven on central NOMAD. Orchestration expertise required for large institutional deployments.

Out-of-the-box readiness	Data is readily available for downloads. Uploading the data required collaboration with the developers.	Easy script-based setup. Most offered functionalities come out of the box. Plugins can be added for extending functionality.	Quickly deployable with docker-compose. All core ELN features enabled by default.	Unclear.	Quickly deployable with docker-compose. Build-in analysis blocks for multiple characterization techniques available by default. New instrument types require custom development.	Each deployment requires hardware-specific SiLA2 server development, NixOS configuration, and custom workflow scripting.	Installable package that comes with dashboard GUI, simulation mode, and base classes. Requires lab-specific development for device drivers and task definitions.	Quickly deployable with docker-compose. Strong built-in support for computational data. ELN/experimental use requires custom plugin development to extend basesections or community schemas.
Provenance depth	Captures synthesis, post processing, and measurement metadata. No semantic provenance graph, no input / output linkages, no experimental - computational cross-linking.	Rich record-linkage provenance for capturing workflow lineages. No native experimental - DFT cross-linking.	Timestamps, revision history, and immutable locking with e-signatures. No native support for analytical or computational provenance.	Preserves directed object-links representing library - measurement - composition chains. No integration with formal provenance standards.	Strong sample provenance connecting samples, batches, and derivatives. Interactive visualization of network graph. No native experimental - DFT cross-linking.	Strong operational provenance: instrument commands, BO decisions, and DFT results. No sample provenance.	Strong operational provenance via DAG task graphs and persistent sample position history. Low sample provenance.	Detailed processing and sample provenance: BFO-inspired basesections, domain-specific community schema, and lab-specific extensions. Provenance tracking with linked entries and workflow graph visualizations. Supports experimental - DFT cross-linking.
Spatially resolved	Uses a 4×11 grid of 44 positions per library substrate. All measurements are spatially registered to shared grid coordinates.	Coordinates can be stored as custom record properties. No system-level spatial coordinate model.	Spatial metadata can be defined in templates as free text or custom properties. No system-level spatial model or viewer.	System-level support for linking measurements to spatial grid positions on library.	No native spatial data model or coordinate registration system.	No native spatial data model or coordinate registration system.	Built-in lab-logistics sample position tracking (for robotic operations). No native support for scientific spatially resolved data.	System-level schema for spatial registration of measurements at grid positions. Multiple measurements shared same set of coordinates. **(Implemented as part of this work)**

Sample model	System-level model where sample is implicitly a position on a combinatorial substrate.	Sample records can be configured using user-defined templates. No system-level sample model.	"Resources" module supports user-level definition for samples and reagents; linkable to experiments. No system-level model.	System-level models for sample and libraries with support for positional linkages.	System-level sample model available. Custom plugin development required for combinatorial libraries and positional linkages.	No system-level or user-level model for samples. Tracked as SQL rows within campaign-specific schemas.	Light-weight system-level sample model with possible extension via MongoDB flexible schema. No formal sample ontology used.	System-level sample model linking with a position on the combinatorial library.
Custom parsing and data normalization	Hardcoded at system-level into the RDI-ETL stack.	User-level parsing and normalization via KadiStudio workflow nodes or external scripts connecting via API.	No native parsing infrastructure. External scripts handle normalization and push structured metadata via the API.	No native parsing or normalization infrastructure.	Plugin-based data block system for custom parsing and normalisation.	No native parsing or normalization infrastructure. Can be implemented in external Python scripts.	Supports task-level Python coding for data pre/post-processing and Pydantic I/O validation.	Plugin-based injection of parsers and normalizers. Normalization coupled with schemas for derived quantities, cross-references, and visualizations.
Analysis write-back	COMBIgor, their analysis package, enables analysis of retrieved data. But write-back of derived results into the repository is not implemented.	New linked records/files or updates on existing records writable via API. No notebook integration or result validation.	Experiment entries, resource records, and files writable via API. No notebook integration or result validation.	New linked objects, result files, or updates on existing records writable via API. No notebook integration or result validation.	Supports built-in structured write-back via data blocks. External write-back via API and datalab-api Python client. No notebook integration.	Native closed-loop write-back for experimental campaigns. No standalone API for analysis write-back outside of active campaigns.	Native closed-loop write-back for experimental campaigns. Per-task result dictionaries stored in database without data validation.	Structured analysis results written back as new entries or updating existing ones via API. Support for integrated analysis notebooks for on-the-fly custom analysis.
Public FAIR export	Public web database with API access and CSV/JSON downloads. No support for schema export or, lowering data interoperability.	Supports export in standard RO-Crate and RDF formats to preserve linkages in data; direct Zenodo publishing is possible.	Supports export in the .eln (based on the RO-Crate specification), PDF, ZIP, CSV, and JSON.	Access levels, search APIs, and shareable search URLs enable structured, role-aware data discovery. No explicit FAIR-standard export documented.	Supports data export in JSON via API. No support for schema exports documented.	Experimental and simulation data reside in a SQL backend, accessible only via direct database queries or custom scripting.	Data stored as JSON in database. No built-in FAIR export.	JSON export for both Metainfo schema and data entries via API. Bundle ZIP via GUI.

S3. Data Model

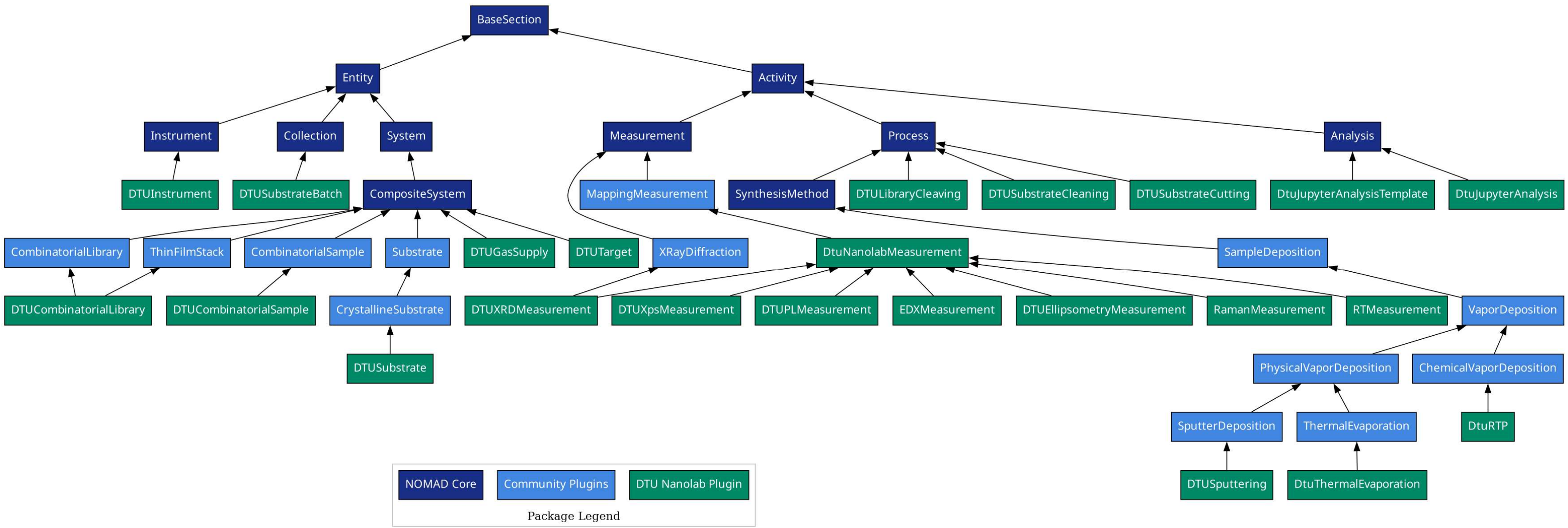

Figure S1. The primary data classes introduced in the `nomad_dtu_nanolab_plugin` (green) and how they inherit from classes in community plugins (light blue) and the core nomad package (dark blue).

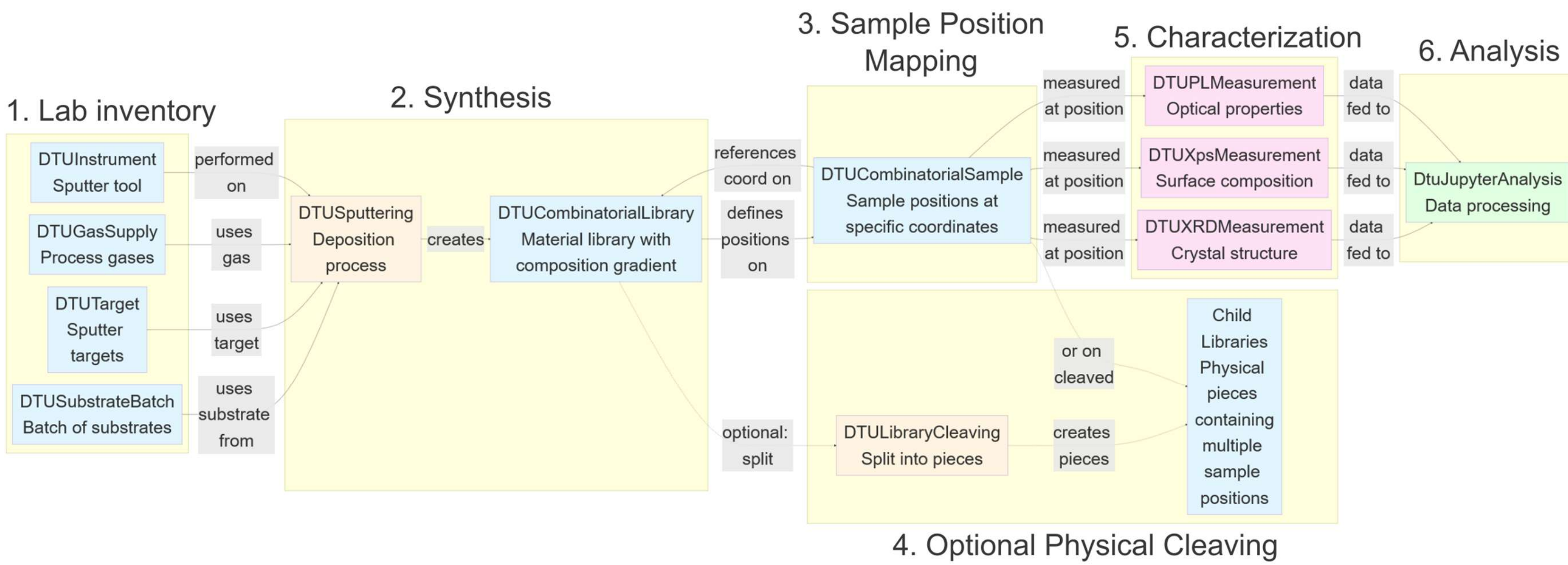

Figure S2. End-to-end workflow illustrating the data classes and their relationships within the NOMAD Oasis. Physical inventory items (1) are linked to the synthesis processes in which they are used (2), which generate combinatorial material libraries. Sample positions on these libraries are mapped in a shared coordinate system (3), optionally followed by physical cleaving into child libraries (4). Characterization measurements acquired at defined sample positions using different techniques (5) are then associated with the corresponding samples and finally processed through integrated, cloud-based analysis workflows (6).

S4. Analysis python notebooks

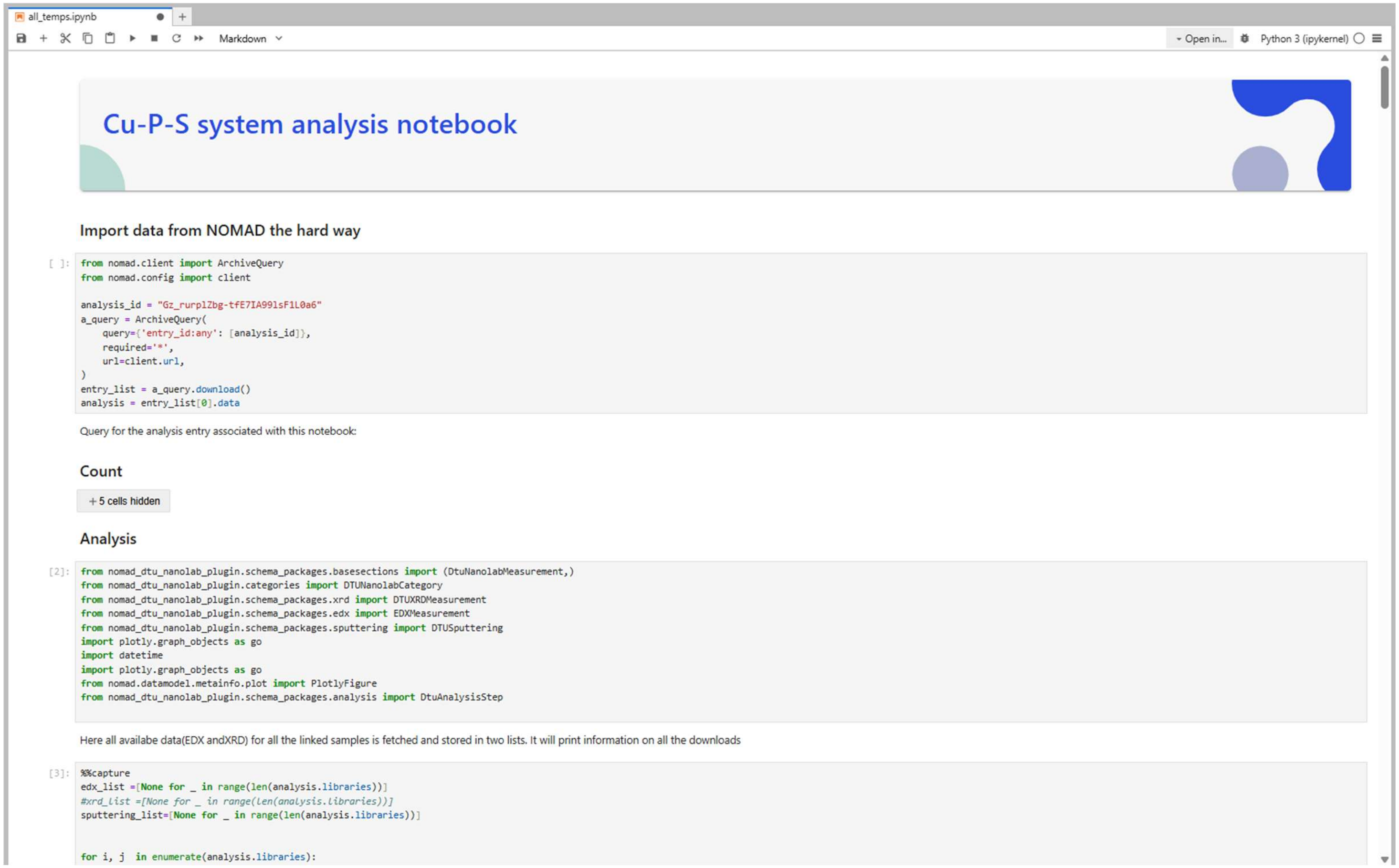

Figure S3. Example of an analysis Jupyter notebook that runs in the Jupyter Hub in NOMAD Oasis.

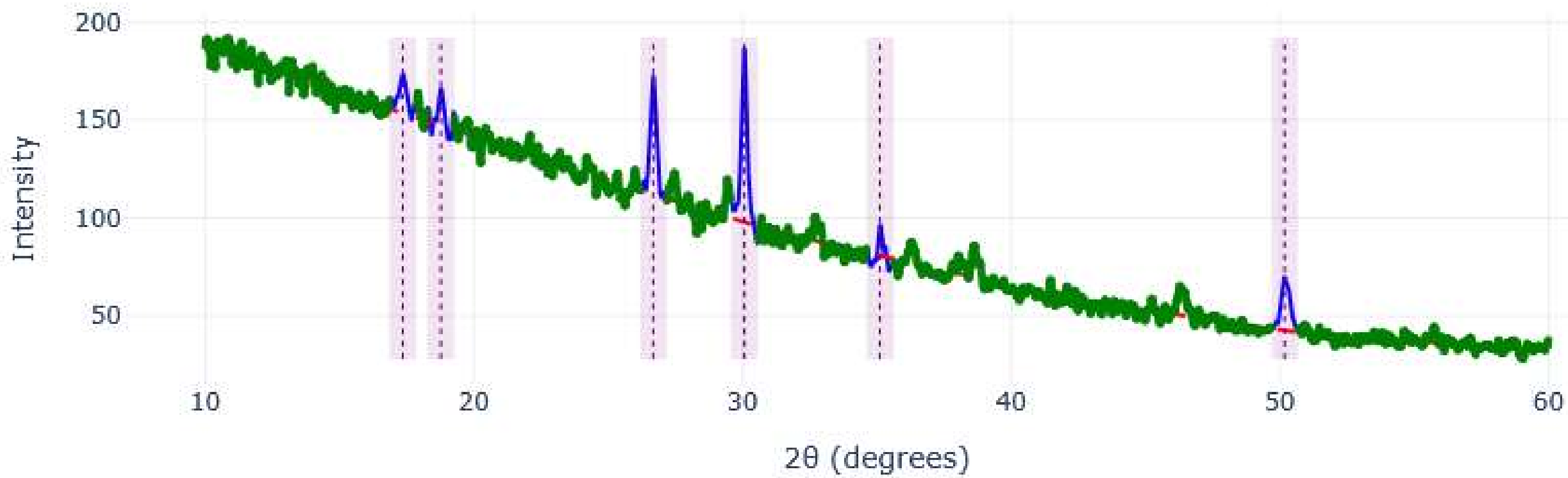

Figure S4: Experimental XRD patterns are loaded using programmatic querying. Post treatment like background fitting and background subtraction can be performed prior to other analysis.

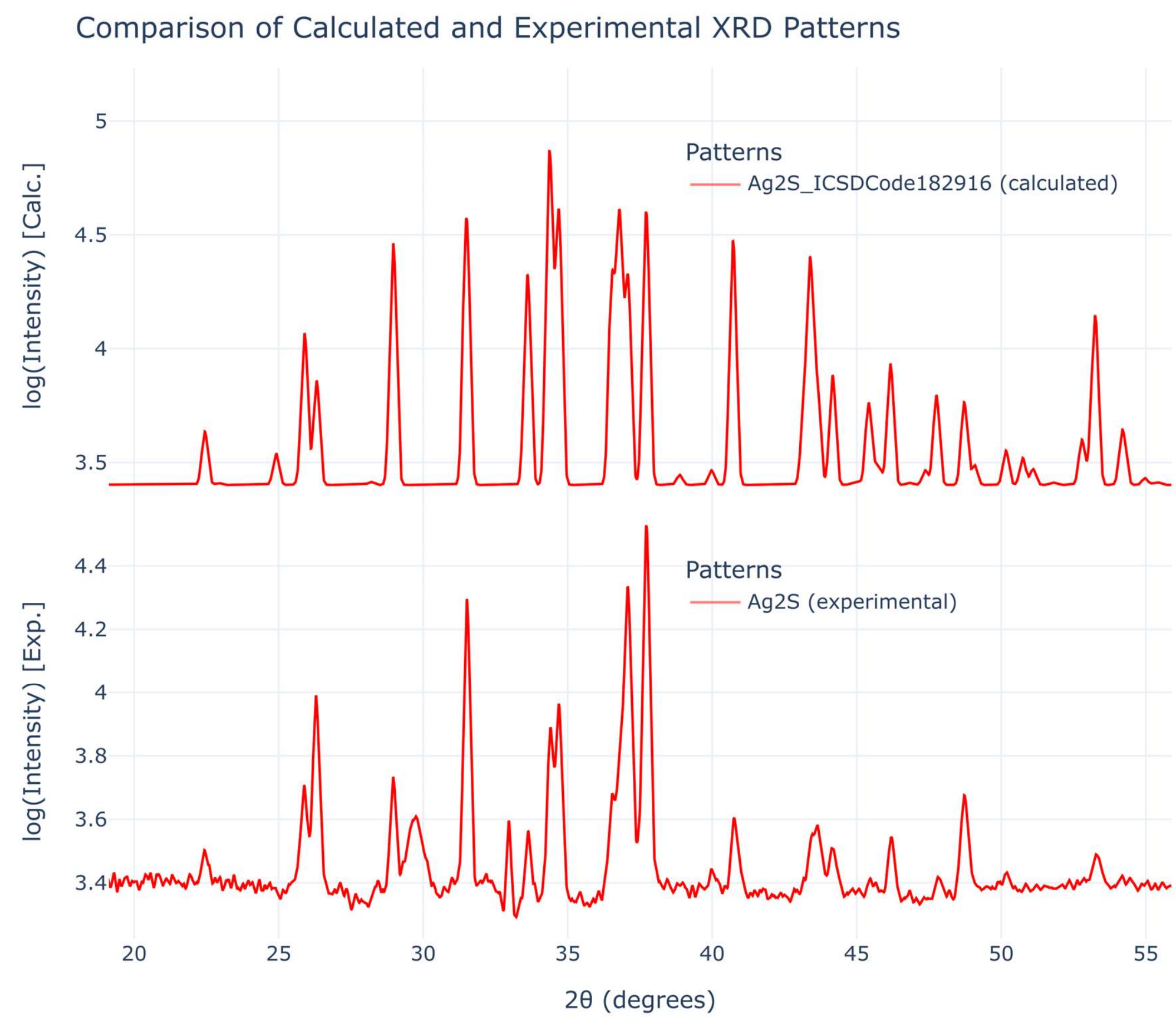

Figure S5. Example figure generated using customized python Jupyter analysis notebooks. Experimental XRD patterns are loaded using programmatic querying and calculated XRD patterns are computed from reference crystals using pymatgen. Calculated patterns (top panel) and experimental patterns (bottom panel) are plotted and compared.

S5. Search Applications

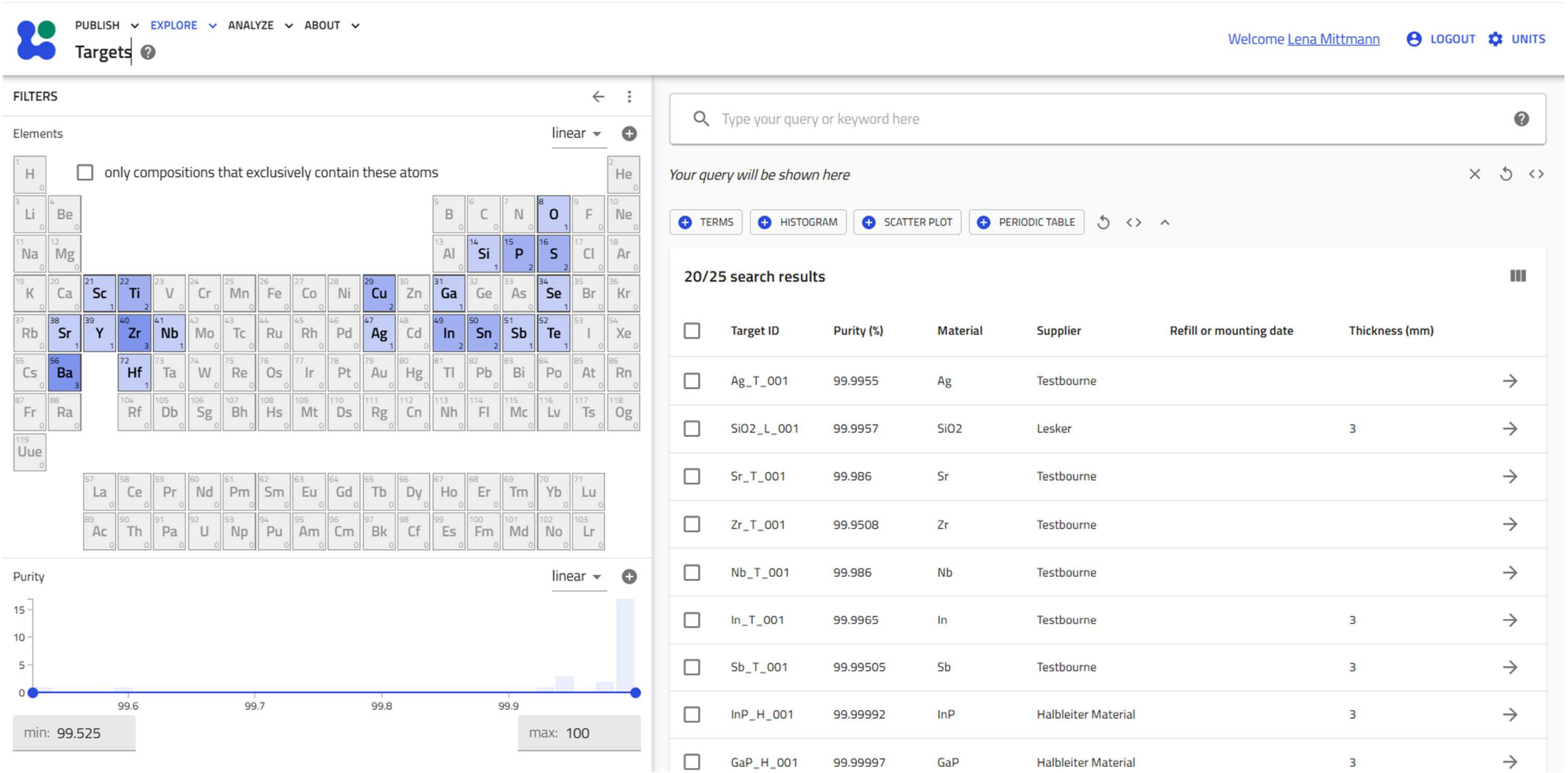

Figure S6. Search interface for sputter targets. Targets can be filtered based on their composition (left panel). Information about target history and impurities can be accessed when opening a specific target entry (right panel)

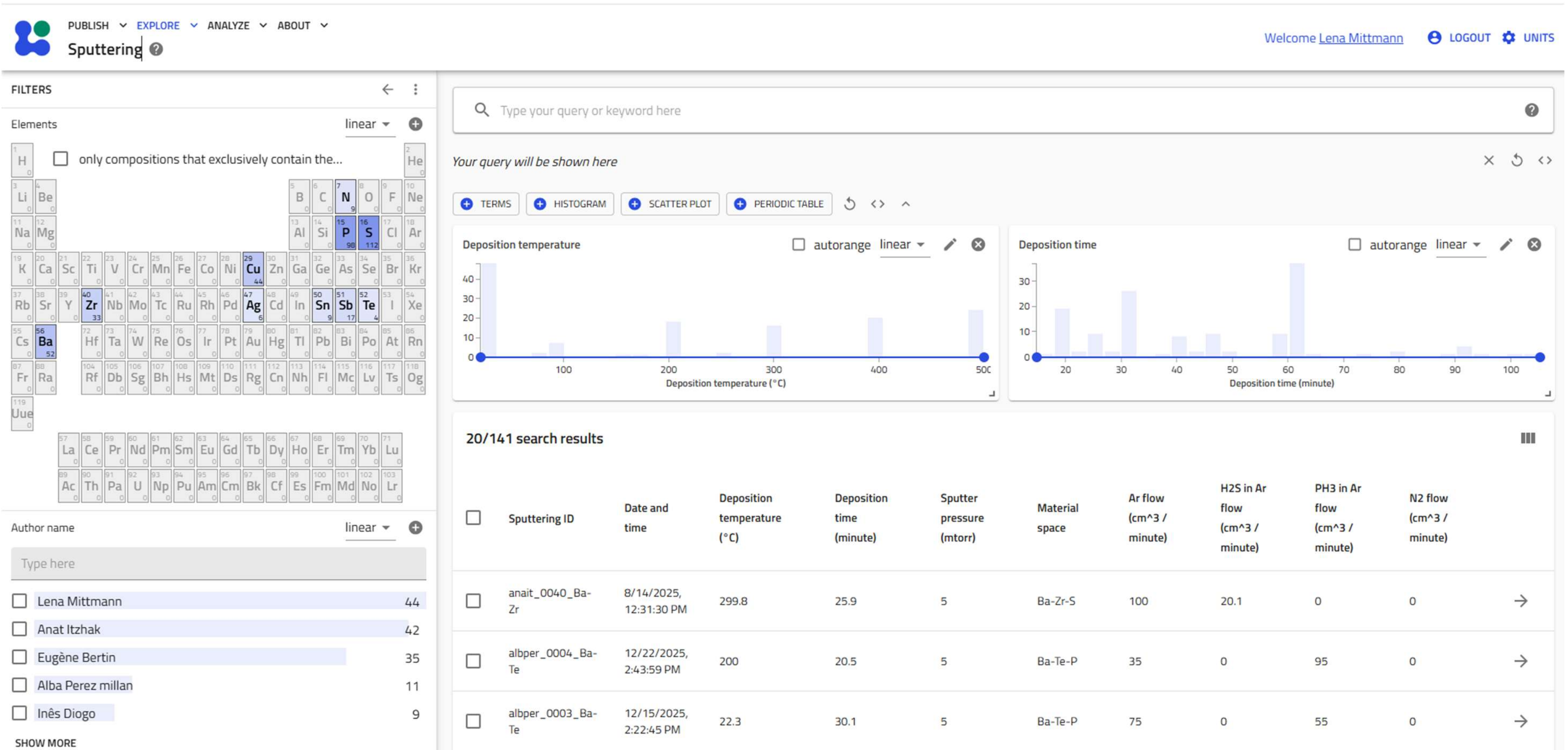

Figure S7. Search interface for sputter deposition processes. Sputtering runs can be filtered based on the elements nominally present on the films (left panel). Information about specific sputtering runs (deposition temperature and duration, gas flows, etc.) can be accessed when opening a specific sputtering entry (right panel)

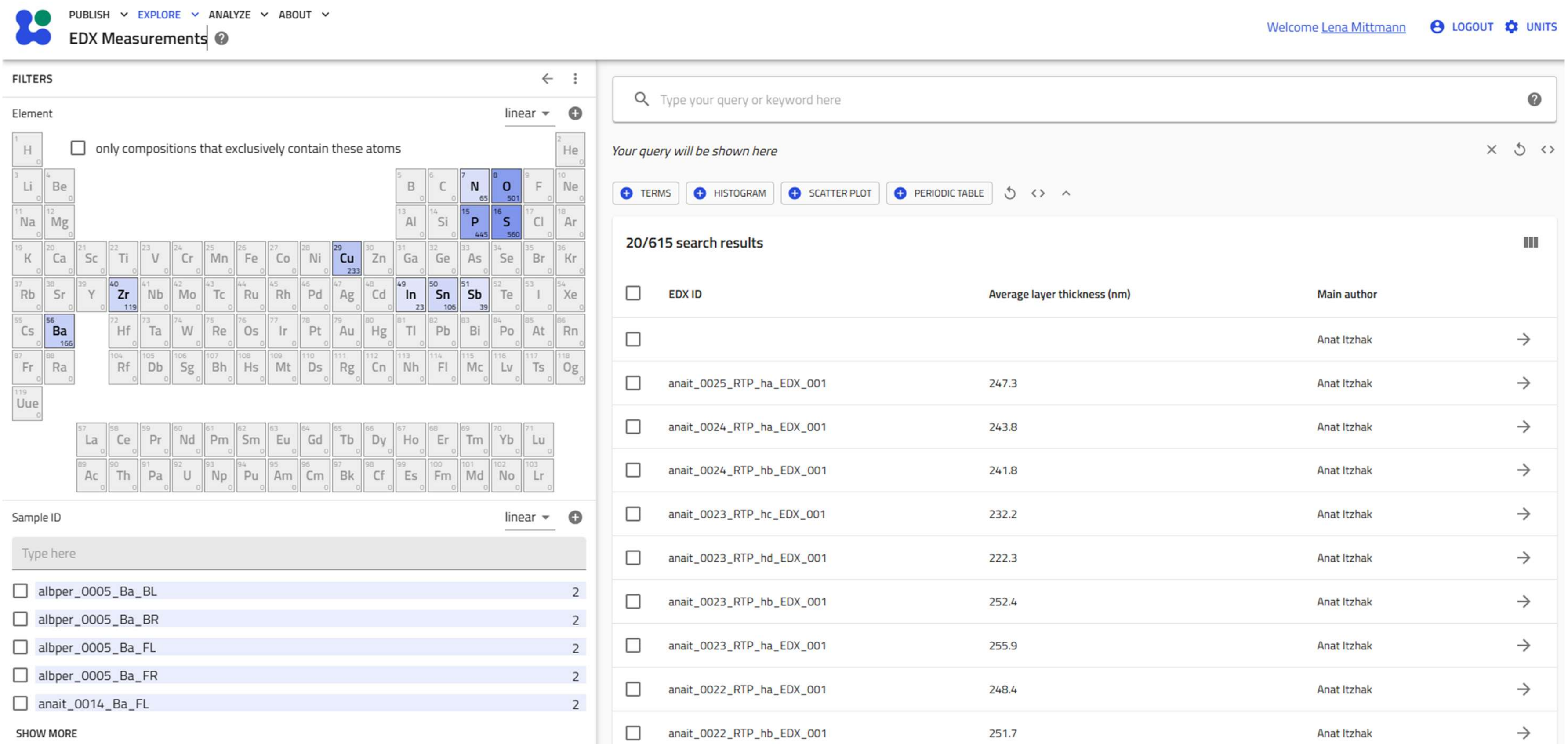

Figure S8. EDX data search interface to find elemental composition of thin films. EDX measurements can be filtered based on detected elements (left panel) and accessed on through right panel.

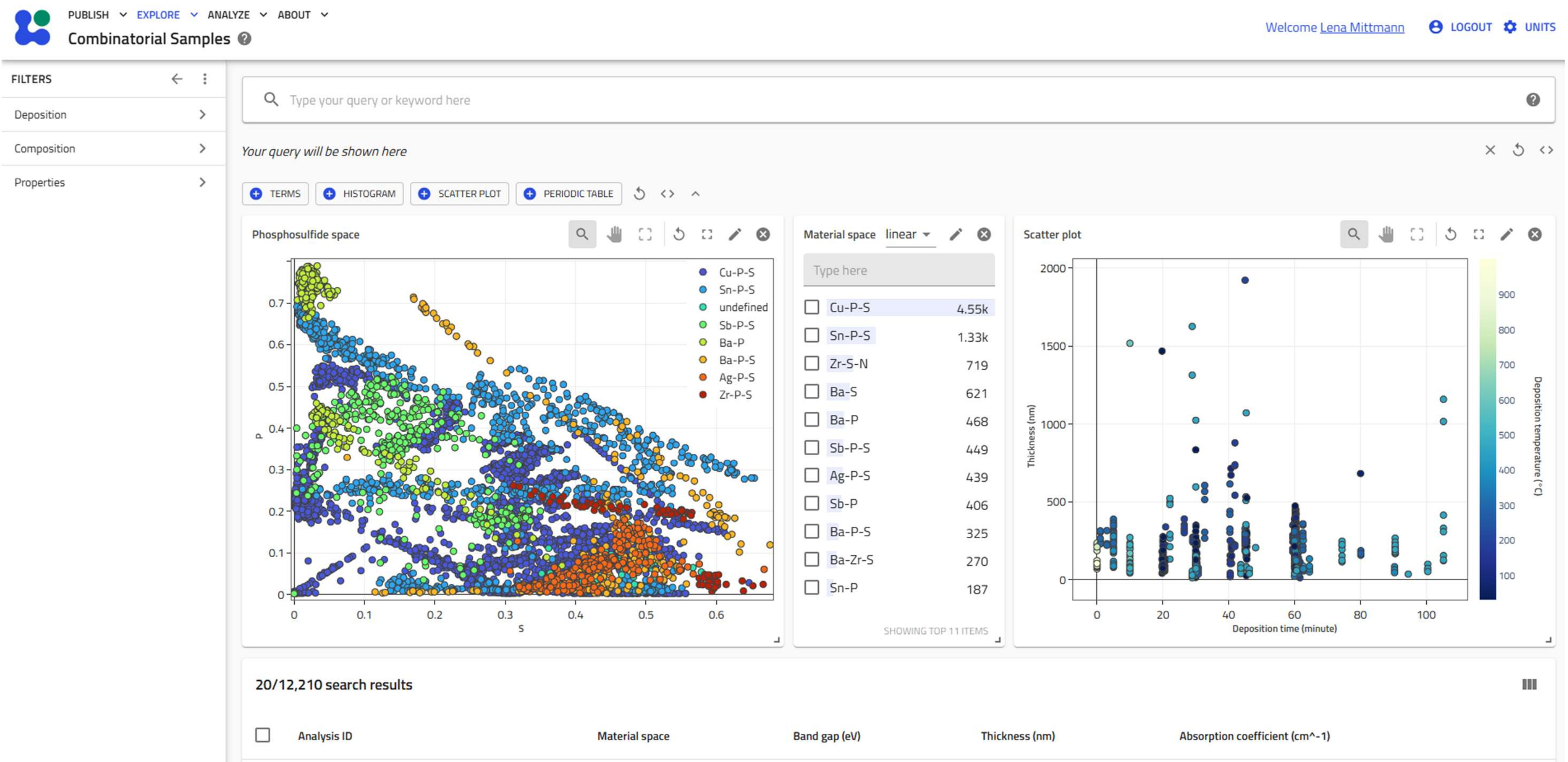

Figure S9. Search application for combinatorial samples.

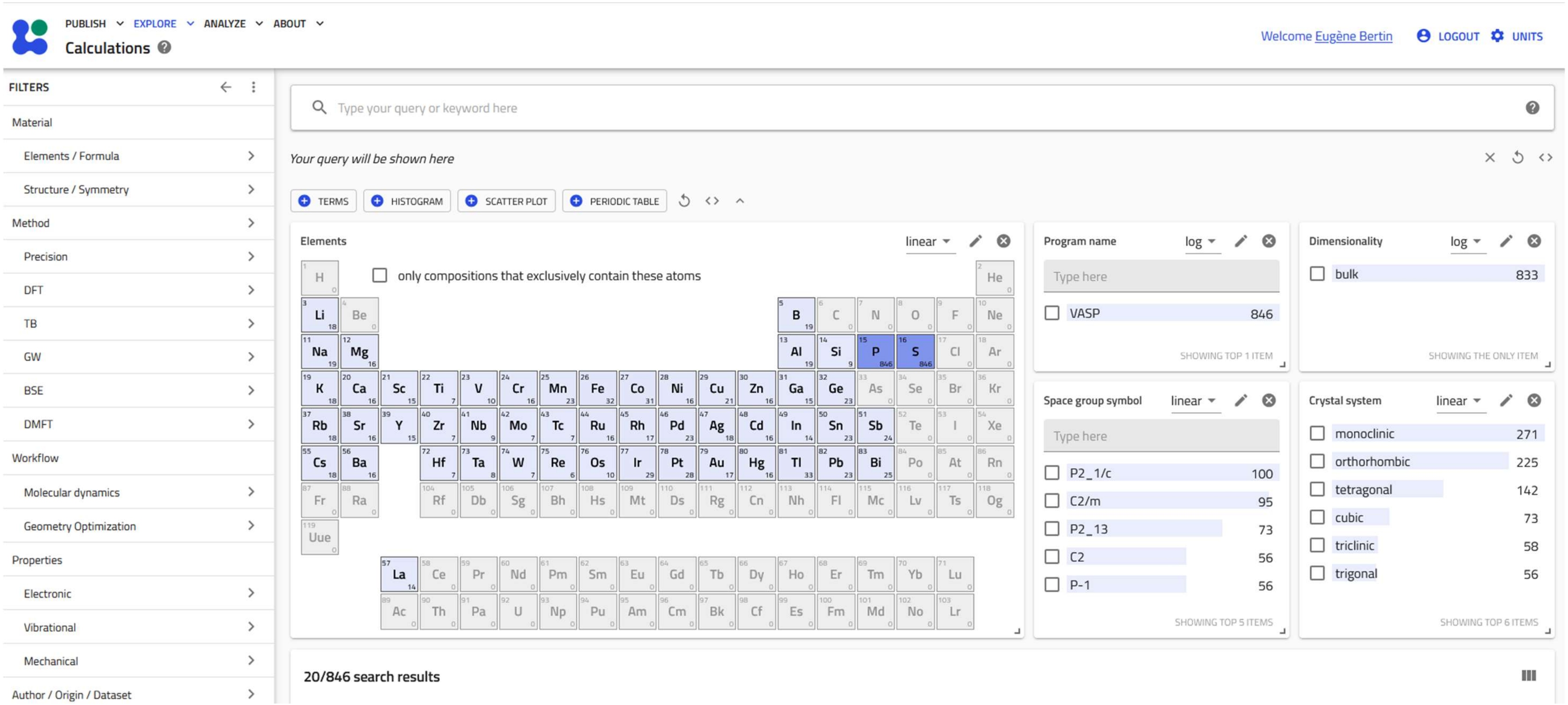

Figure S10. Search application for DFT calculations. DFT entries can be filtered based on included elements (left panel) or space group, crystal system or other characteristic and reported properties for the respective entry.

S6. Additional automatically generated plots

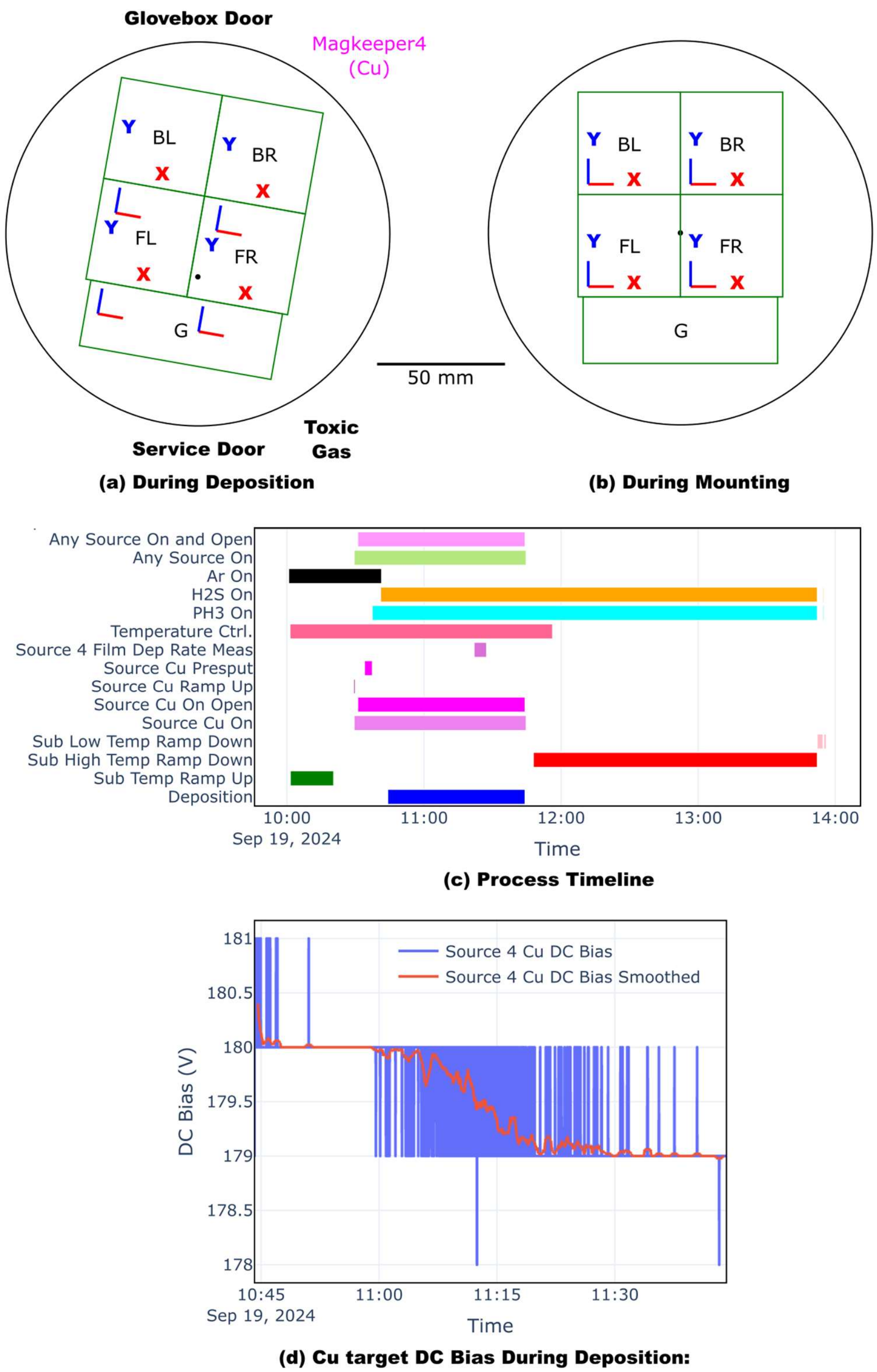

Figure S11. Default graphs obtained from parsing a sputter process log file through the sputtering entry, as automatically visualized on our NOMAD Oasis. (a) Position of the different substrates on the platen during deposition, relative to the sources. The sources are a reactive toxic gas inlet (H_2S + PH_3), a sputter target (here, Cu) and other sputtering chamber landmarks. (b) Position of the substrates during mounting, defining the orientation of base coordinate system for the combinatorial library. (c) Process timeline graph representing different process-relevant events,

such as the moments where reactive gases were flown in the chamber (PH_3 On, H_2S on) or the moment of the deposition itself. (d) Time trace of the DC self-bias developed by the Cu target during the deposition.

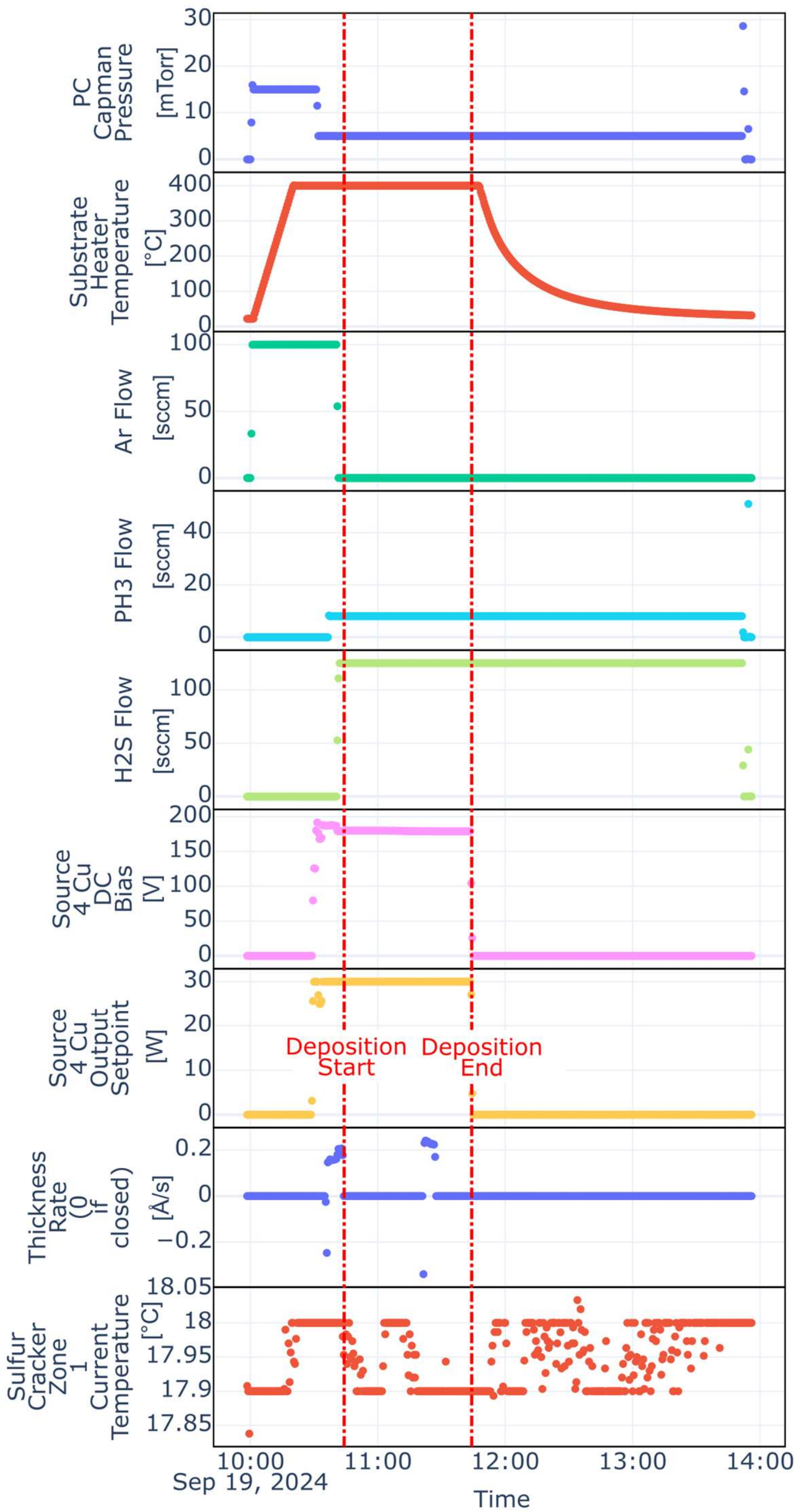

Figure S12. Overview plots of multiple sputtering process signals like gas flow rates and substrate temperature, before, during and after the deposition, spanning the entire process.

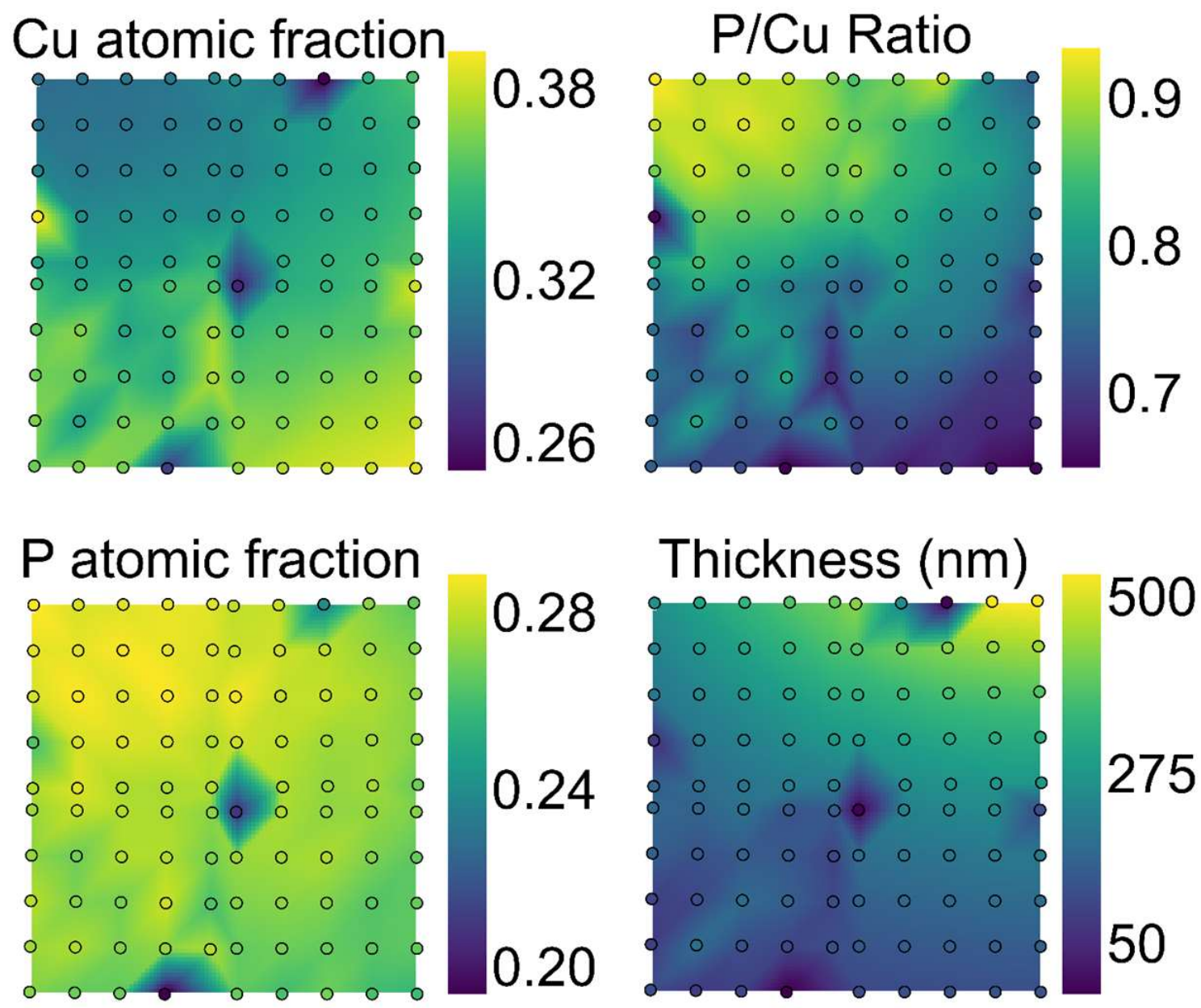

Figure S13. EDX map showing atomic fraction, thickness and an element ratio plot as provided after uploading the respective data.

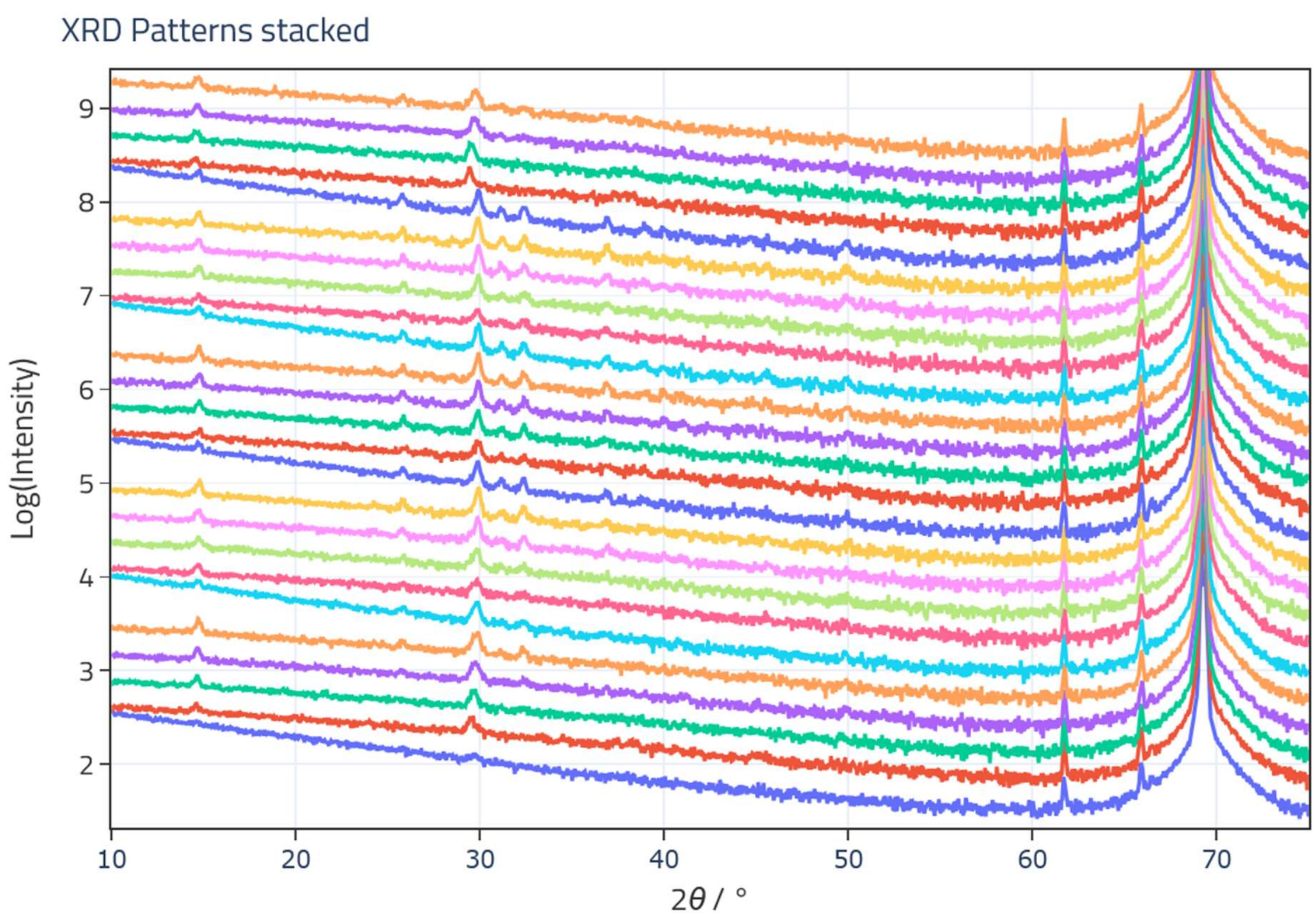

Figure S14. XRD stacked plot for a mapping measurement on a combinatorial library.

S7. Limitations, considerations, and learnings

Infrastructure needs:

- A suitable server (RAM, storage, backup)
- Backup procedures and capacity for backup files
- Allowance to use the university servers to host a **public** database
- Infrastructure to test the developments before deploying updates (remote computer, laptops with sufficient capacity and computing power)
- Someone who knows about server architecture and how to operate and maintain a website on the university servers

Development considerations:

- Need for a combination of coding skills and experimental skills; AI coding tools help speed up development and make coding more accessible to experimentalists
- High knowledge (experimental and coding) requirements in an environment where 2–3-year time-limited contracts are commonplace
- Need to establish and teach Git(Hub) procedures and etiquette
- Coding knowledge is also required during analysis (the whole group needs to learn more coding, even if not actively developing the infrastructure)
- Mentoring from NOMAD developers (takes time and capacity from both sides, especially in the beginning)

Updating tasks:

- Maintaining the database and spotting incompatibilities
- Error handling after (re)processing runs
- Finding and fixing duplicates, faulty entries, and broken references
- Keeping up to date with new developments at central NOMAD
- Adapting to new requirements (flexible and not restricting)
- Establishing user feedback loops to improve usability
- Ensuring retro compatibility for old experiments and previous worflows

Documentation suggestions:

- Extensive documentation for development procedures, which helps ensuring the continuity of development
- “How-to-use schemas” and “how-to-create entries and uploads” documentation
- Trouble shooting manual (fixes and tricks) for common errors
- Continuous maintenance of such documentation

Data structure and querying limitations:

- Nested structure makes API access of data harder when unfamiliar with the exact structure
- API documentation is not sufficient for unexperienced API users
- References go only one way; There is a need to know how to query for the back references
- User interface access is difficult when you are completely unfamiliar with the data structure and data
- Search interfaces need to be created for more intuitive access
- Visibility of data and references is limited to shared data, which can lead to confusion when the data sharing is not done correctly

Transferability considerations:

- Choices in implementation (flexibility vs comfort): what works for one does not work for another person
- Limited changes in the data schemas
    - no deleting of editable or non-editable properties
    - Adding properties requires reprocessing of existing uploads
- future-proof schemas that offer the most convenience and flexibility simultaneously
- Ideally, we should use this effort to create community standards, but that is challenging due to the different requirements. Our solution is not an out-of-the-box solution for all other groups, and a certain level of customization will always be necessary
